# Supplementary material for: CELEB trial: Comparative Effectiveness of Lung volume reduction surgery for Emphysema and Bronchoscopic lung volume reduction with valve placement: a protocol for a randomised controlled trial
Source: BMJ Open. 2018 Oct 17;8(10):e021368. doi: 10.1136/bmjopen-2017-021368 (PMC6196851; doi:10.1136/bmjopen-2017-021368)
Supplement: Author's manuscript [file bmjopen-2017-021368.draft_revisions.pdf]

BMJ Open is committed to open peer review. As part of this commitment we make the peer review history of every article we publish publicly available.

When an article is published we post the peer reviewers' comments and the authors' responses online. We also post the versions of the paper that were used during peer review. These are the versions that the peer review comments apply to.

The versions of the paper that follow are the versions that were submitted during the peer review process. They are not the versions of record or the final published versions. They should not be cited or distributed as the published version of this manuscript.

BMJ Open is an open access journal and the full, final, typeset and author-corrected version of record of the manuscript is available on our site with no access controls, subscription charges or pay-per-view fees (http://bmjopen.bmj.com).

If you have any questions on BMJ Open's open peer review process please email info.bmjopen@bmj.com

BMJ Open

The CELEB trial: Comparative Effectiveness of Lung volume reduction surgery for Emphysema and Bronchoscopic lung volume reduction with valve placement: a protocol for a randomised controlled trial

Journal:	*BMJ Open*
Manuscript ID	bmjopen-2017-021368
Article Type:	Protocol
Date Submitted by the Author:	02-Feb-2018
Complete List of Authors:	Buttery, Sara; Royal Brompton and Harefield NHS Foundation Trust,<br>Kemp, Samuel; Royal Brompton and Harefield NHS Foundation Trust<br>Shah, Pallav; Royal Brompton and Harefield NHS Foundation Trust<br>Waller, David; Barts Health NHS Trust<br>Jordan, Simon; Royal Brompton and Harefield NHS Foundation Trust<br>Lee, John; National University of Singapore,<br>Banya, Winston; Royal Brompton and Harefield NHS Foundation Trust<br>Steiner, Michael; Institute for Lung Health,<br>Hopkinson, Nicholas; Royal Brompton Hospital, Respiratory Muscle Lab
Keywords:	COPD, Lung Volume Reduction, Thoracic surgery < SURGERY, Emphysema < THORACIC MEDICINE

SCHOLARONE™ Manuscripts

For peer review only

The CELEB trial: Comparative Effectiveness of Lung volume reduction surgery for Emphysema and Bronchoscopic lung volume reduction with valve placement: a protocol for a randomised controlled trial

[1]Sara Buttery, [1]Samuel V. Kemp, [1]Pallav L. Shah [3] David Waller, [1]Simon Jordan, [4]J L. Lee,

[1]W. Banya, [2]Michael Steiner, *[1]Nicholas S. Hopkinson

[1]NIHR Respiratory Disease, Biomedical Research Unit at the Royal Brompton and Harefield NHS Foundation Trust and Imperial College London, London, UK

[2] Leicester Respiratory Biomedical Research Unit, University Hospitals of Leicester NHS Foundation Trust, Leicester, UK

[3] Barts Health NHS Trust London, London, UK

[4] National University of Singapore, Singapore, SG119260

Corresponding author: S.Buttery

Royal Brompton Hospital

Fulham Road

London

SW3 6HP

Email: s.buttery@rbht.nhs.uk

Telephone: 0207 351 8029

Fax: 0207 351 8939

For peer review only

Abstract:

Introduction: Although lung volume reduction surgery and bronchoscopic lung volume reduction with endobronchial valves have both been shown to improve lung function, exercise capacity and quality of life in appropriately selected patients with emphysema, there are no direct comparison data between the two procedures to inform clinical decision making.

Methods and Analysis: We describe the protocol of the CELEB study (**ISRCTN**19684749), a randomised controlled trial which will compare outcomes at 1 year between the two procedures, using a composite disease severity measure, the iBODE score, which includes body mass index, lung function, breathlessness and exercise capacity.

Ethics and Dissemination: Ethical approval to conduct the study has been obtained from the Fulham Research Ethics Committee, London (16/LO/0286). The outcome of this trial will provide information to guide treatment choices in this population and will be presented at national and international meetings and published in peer-reviewed journals. We will also disseminate the main results to all participants in a letter.

Strengths and Limitations

- This is a randomised controlled trial
- There is a strict patient selection criterion for this trial based on previous research.
- Currently there is equipoise between the two interventions being compared.
- Due to the nature of the interventions it is not possible to double blind the trial.
- It may be difficult to obtain longer term outcomes as patients may cross over after the 12 month period of the trial.

For peer review only

Introduction:

Chronic obstructive pulmonary disease (COPD) is a common and disabling condition which is now the third largest cause of death worldwide[1]. Breathlessness, exercise limitation and mortality in COPD are all associated with increased lung volumes occurring due to airflow obstruction and increased lung compliance[2]. The condition is progressive and despite optimum care including smoking cessation, pharmacotherapy and pulmonary rehabilitation, many patients remain breathless and limited in everyday activities[3 4]. Surgical and bronchoscopic approaches to lung volume reduction are available which can bring substantial benefits in appropriately selected individuals, though both are also associated with some risk.

Lung volume reduction surgery (LVRS) involves removing the worst affected area of emphysematous lung, which means that the remaining healthier and less compliant lung can function more effectively, with the respiratory muscles working at less of a mechanical disadvantage[5]. LVRS has been shown to improve survival, exercise capacity and quality of life in appropriately selected patients with heterogeneous emphysema and poor exercise capacity [5-9] and is therefore recommended in national and international guidelines for the management of COPD.[2 10 11] However, uptake has been limited, due in part to exaggerated concerns about surgical morbidity and mortality[12]. In modern clinical practice morbidity and mortality from the procedure is substantially lower[9 13] than was the case in trials conducted around the turn of the century.[5 6]

An alternative approach to resecting the target area of lung is to place endobronchial valves occluding the airways supplying a particular lobe. This form of bronchoscopic lung volume reduction (BLVR) can produce lobar atelectasis, and is intended to achieve similar benefits to LVRS but with less morbidity[14-24]. The approach is only effective in the absence of interlobar collateral ventilation (CV)[24 25]. If this is present air can enter the target lobe from an

For peer review only

adjacent lobe and atelectasis does not occur. In patients with a heterogeneous pattern of emphysema and intact interlobar fissures, valve placement can produce significant improvements in lung function, exercise capacity and health status.[24 26-28] Yet complications do occur, in particular pneumothorax which occurs in up to 30%of cases[10 16 26] and can on occasion be fatal, as well as exacerbation-like events. Valve expectoration or misplacement can necessitate additional procedures.

Patients with heterogeneous emphysema and intact interlobar fissures, may benefit from either BLVR or LVRS, but there are no direct comparison data on the relative value of the two procedures to guide clinical decision making.

The CELEB study is a multi-centre, randomised - controlled parallel group trial. Patients, who are considered to be suitable candidates for both forms of targeted lung reduction therapy, will be randomised to either BLVR or LVRS. The primary aim of the CELEB study is to determine whether LVRS produces a greater health benefit at one year than BLVR. This will be determined using change in the iBODE index, a composite measure of COPD severity (made up of BMI, lung function, breathlessness and exercise capacity).[29 30] Change in BODE score has been found to be a good predictor of survival in LVRS patients[31]. Health resource utilisation will also be compared between treatment arms. Clinical efficacy will be assessed over a 12 month period. The study is set up as a superiority trial and will be conducted, analysed and reported according to the Consolidation Standards of Reporting Trials (CONSORT) statement for randomised controlled trials. This paper will outline the protocol for the CELEB study (version 2.0, 19th February 2016).

Methods and Analysis:

Participants: Patients will be recruited from outpatient clinics at UK hospital sites which have an established multi-disciplinary team (MDT) meeting dedicated to identifying suitable

candidates for LVR[32]. Patients will be eligible for the study if they fit the following criteria; significant airflow obstruction (FEV_1<60% predicted), limiting breathlessness (MRC dyspnoea score > 3), significant hyperinflation (TLC>100% predicted, RV>170% predicted) and are considered, using CT and perfusion scan, to have a heterogeneous emphysema pattern with intact interlobar fissures (>90%). They must also not have smoked for at least 3 months. Patients will be excluded if they have pulmonary fibrosis or any other major co-morbidity that could affect survival or mean that LVR procedures are unlikely to be effective. Patients with PaO_2<7.0kPa and/or $PaCO_2$>7.0kPa will also be excluded.

Interventions: LVRS will be carried out by a thoracic surgeon under general anaesthetic using a unilateral VATS approach. The precise operation will be at the discretion of the surgeon, intended to remove the most emphysematous area of lung, leaving the best quality lung behind and minimising air leaks and other complications. Patients will initially go to HDU post operatively and post-operative management will include attention to wound discomfort, management of chest drains and prompt mobilisation. Patient management, discharge and initial follow up will be determined by their clinical team.

BLVR will be performed via bronchoscopy by an operator experienced in placing EBV. The procedure will be performed under conscious sedation or general anaesthetic if necessary, with endobronchial valves placed to occlude the target lobe. A chest x-ray will be performed one hour post procedure and participants will spend three nights post-procedure as an in-patient routinely in case a pneumothorax occurs. They will be discharged with written advice about the signs of pneumothorax and what to do if these occur. Procedures including valve adjustment and replacement will be permitted to ensure treatment is optimised.

Participants will be followed up in clinic routinely about three months after discharge. Other clinic attendances will depend on clinical need with further investigations and procedures determined by their responsible clinician. Patients will receive a phone call from the study

For peer review only

team at one month and at 6 and 9 months. There will be a final clinic visit at 12 months. A summary of the trial assessment is shown in **Table 1.**

Outcomes: The Primary outcome will be the change in iBODE score[37] at 12 months from baseline. The iBODE incorporates the incremental shuttle walk test, BMI, FEV_1 and the MRC dyspnoea score. Possible scores range from 0 to 10 with 10 with increasing scores associated with worsening mortality. The primary endpoint measures will be performed by staff blind to treatment allocation and patients will be asked not to reveal this in order to reduce bias.

Secondary outcomes will be assessed at baseline, 3 months and 12 months. Changes in health related quality of life (CAT score), residual volume (RV) and fat free mass (FFM) will be evaluated. Physical activity will be measured using the Proactive c-PPAC score.

The basis for the sample size calculation is the paper by Imfeld *et al* which addressed the response to LVRS [31]. This compared the improvement in BODE score at three months post LVRS between survivors and non-survivors at 5 years. Long term survivors had an improvement of 3.9 points at three months, the non–survivors, had an improvement of 2.4 points (*i.e.* a difference of 1.5 points). This difference is likely therefore to represent a clinically important difference between outcomes in the two LVR arms, being of sufficient magnitude to influence treatment decisions and is therefore a meaningful basis on which to power the study.

The study is powered on the basis of an average response to LVRS that will be the same as in the Imfeld paper (*i.e.* a fall of 3.1) and for BLVR that the improvement will be 1.6 (1.5 points less benefit). Assuming an SD of 1.8 for the change in BODE score and taking a 5% significance level and 90% statistical power, 34 patients would be required in each treatment arm. Allowing for 10% drop-outs we will enrol 76 individuals

For peer review only

Assignment of interventions: All participants will undergo a fibreoptic bronchoscopy to allow for assessment of the presence of collateral ventilation using the Chartis^{TM} system[25]. This uses a balloon catheter, inflated in the target airway, with a flow sensor and a distal pressure meter. Pressure swings should continue with inspiration and expiration, but flow will diminish and then cease as the target lobe empties if there is no collateral ventilation (CV –ve). If collateral ventilation is present, flow will continue (CV +ve). Subsequent participation in the trial depends on this. As the absence of collateral ventilation in the target lobe is an inclusion criteria patients with collateral ventilation are deemed to be screen failures and cannot proceed to randomisation (as they would not benefit from valve placement so there is no longer equipoise). If these patients do go on to "open label" LVRS their iBODE score will be evaluated 12 months following that procedure. In some cases CV cannot be assessed either because there is continuing low flow or an abrupt cessation in the flow trace suggesting obstruction. It may be possible to work round this by measuring the presence or absence of CV in the adjacent lobe (*i.e.* the reciprocal position). Where CV is truly indeterminate, patients will be withdrawn from the study (*i.e.* treated as CV positive).

If the person has a CV negative lobar target for treatment they will be immediately allocated randomly to one of the treatment arms, using an online system. Randomisation will be on a 1:1 basis based on a computer generated random sequence with random block sizes generated by Sealed Envelope ltd (London, UK). This will include stratification by site and by iBODE score to ensure matched numbers in both arms with an iBODE score of ≥ 7.

Patients, the study co-ordinator and those providing clinical care will not be blind to treatment allocation. The assessors recording the components of the iBODE score will be blinded to reduce the potential for bias. Therefore no un-blinding process will be necessary.

For peer review only - http://bmjopen.bmj.com/site/about/guidelines.xhtml

Data collection, management and analysis:

At baseline a full medical history including exacerbations, hospital admissions and drug history will be taken. Pulmonary Function Tests (spirometry, plethysmography and gas transfer measurements) will be carried out according to the American Thoracic Society/European Respiratory Society recommendations, together with arterial blood gas analysis. Additional measures will include Medical Research Council dyspnoea score, health status (COPD assessment test (CAT) and EQ-5D) and fat free mass using bioimpedance. Two incremental shuttle walk tests (ISWT) will be carried out due to the learning effect seen following a test walk.[33]

Physical activity will be assessed using the PROactive Consortium patient reported outcome tool for physical in COPD[34-36]. Participants wear a Dynaport Movemonitor accelerometer for one week as a measure of physical activity at the end of this they complete the c-PACC physical activity questionnaire which asks about the amount and difficulty of physical activity during the period covered by the monitor.

A cost-utility analysis will be carried out from a National Health Service (NHS) perspective using data from the trial. The relative value of the two approaches will be based on procedural cost, length of stay, days in hospital subsequently expressed as a cost per quality-adjusted life year (QALY) together with data gathered using the Client Socio-Demographic and Service Receipt Inventory (CSRI) which asks participants about their use of health and social services over the last three months. This will be completed at 3, 6, 9 and 12 months. They will be asked to report the frequency and intensity of their service use. Cost incurred in both arms may include (i) medical and surgical services received as an in-patient

For peer review only

(ii) health care visits in relation to COPD (iii) COPD related medication use. Total cost of individuals will be estimated by multiplying resources use with national tariff used in the NHS. QALY will be estimated using EQ-5D questionnaire. The five attributes of this questionnaire (mobility, self-care, usual activities, pain/discomfort/ anxiety/depression) will be summarised into a single UK-derived preference-based utility score. ***Table 1*** summarises the trial assessments that will be carried out at each visit.

Figure 1

The database will be checked for completeness and consistency. Reference ranges will be set prior to the start of the study and queries generated on the data for values which sit outside of the pre-defined range. Frequency distribution checks will be carried out on the data for possible outliers. The numbers (with percentages) of losses to follow-up (defaulters and withdrawals) over the period of follow up will be reported and compared between the randomised groups.

Descriptive statistics will be presented in terms of the numbers and proportions of patients or, for continuous variables, means or medians, range of observations and standard deviations. Demographic and clinical characteristics of patients at baseline will be presented by randomised group. Data will also be presented on the following; number of patients entered into the study, number of patients with follow-up data.

The results of the study will be presented in terms of the numbers and proportions of patients or, for continuous variables, means or medians, range of observations and standard deviations.

For peer review only

Outcome data analysis will be by intention to treat. Patients with missing data will be assumed to have had zero change from baseline. Unpaired t-tests will be used to compare mean response across the 2 groups: control and intervention. Mixed linear model procedure to evaluate the effects of predefined covariables on dependent outcome variables.

Total costs will be calculated as the sum-product of resource use and unit cost for each patient. Incremental cost-effectiveness ratios (ICER) will be obtained by calculating the incremental costs divided by the incremental effects for the LVRS vs the BLVR groups`. Multivariate regression analysis will be used to adjust for baseline differences in health status between the trial groups. Uncertainty in the incremental cost-effectiveness ratio (ICER) will be addressed using bootstrapping and the estimation of cost-effectiveness acceptability curves. Estimates of ICERs will be compared with the £20,000 to £30,000 per QALY threshold of cost-effectiveness recommended by the National Institute for Health and Care Excellence (NICE). No interim analyses are planned and the trial has no formal stopping guidelines.

Monitoring: All serious adverse events (SAE's) will be reported to Fulham Research Ethics Committee (REC) and written up in patients' medical notes. Adverse events (AE's) will not be reported to the REC but will be recorded in the patients' medical notes and case report form (CRF). A trial steering committee will meet quarterly, including representatives from the study sites and an independent chair.

.

For peer review only

.

Ethics and dissemination: Ethical approval to conduct the study has been obtained from the Fulham Research Ethics Committee, London (16/LO/0286)

Patients will receive oral and written information regarding the study and written consent will be taken. Trial procedures will only begin following a clinical decision by the MDT that a patient is eligible for BLVR or LVRS.

Personal information about potential and enrolled participants will be handled in accordance with the Data Protection Act 1998, NHS Caldecott Principles, The Research Governance Framework for Health and Social Care, 2nd Edition (2005), and the condition of the main REC approval. The Case Report Forms (CRFs) will not bear the subject's name or other personal identifiable data. The subject's initials, Date of Birth (DOB) and trial Identification Number (ID), will be used for identification. Access to anonymised study data will be via application to the Chief Investigator, Dr Nicholas Hopkinson. Decisions about continuation or termination of the trial or substantial amendments to the protocol will be the responsibility of the Trial Steering Committee (TSC).

We intend to present the results of the trial at national and international meetings and publish in peer-reviewed journals. We will also disseminate the main results to all participants in a letter. Research papers will be written by the triallists.

For peer review only

Contributorship Statements: This protocol was written by the Chief Investigator (CI) Nicolas Hopkinson (NSH) in accordance with the Sponsor's guidance for writing non-CTIMP protocols. NSH and the Research Office discussed and agreed this study protocol. All other applicants SB, SVK, PLS, DW, SJ, JLL, WB, MS were involved in trial design and revisions of the final protocol and will be involved in the production of the final manuscript upon trial completion. All investigators (NSH, SB, SVK, PLS, DW, SJ, MS) agreed to perform the investigations outlined in the study protocol and to abide by this protocol except in the case

of medical emergency that will be notified to the Research Office. All authors have agreed upon the final manuscript for this submission.

Funding Statement: The study is funded by a grant to Royal Brompton and Harefield NHS Foundation Trust, from the NIHR, through the Research for Patient Benefit scheme (PB-PG-1014-35051).

Competing Interests Statement: No support from any external organisation or financial relationships with any organisations that might have an interest in the submitted work has been sought for this study. Royal Brompton has received reimbursement of clinical trial expenses from PneumRx, PulmonX, Olympus, Uptake Medical, Holaira, and Creo Medical. The sponsor will not be involved in the conduct of the trial or influence any decision to publish.

Trial sponsor representative

Mr Patrik Pettersson

Royal Brompton and Harefield NHS Foundation Trust (RB&HFT)

Royal Brompton Hospital

Research office

Chelsea Wing, Level 2

Sydney Street

London , SW3 6NP

For peer review only

References:

1. Lozano R, Naghavi M, Foreman K, et al. Global and regional mortality from 235 causes of death for 20 age groups in 1990 and 2010: a systematic analysis for the Global Burden of Disease Study 2010. Lancet (London, England) 2012;380(9859):2095-128. doi: 10.1016/s0140-6736(12)61728-0 [published Online First: 2012/12/19]

2. Lozano R, Naghavi M, Foreman K, et al. Global and regional mortality from 235 causes of death for 20 age groups in 1990 and 2010: a systematic analysis for the Global Burden of Disease Study 2010. *Lancet (London, England)* 2012;380(9859):2095-128. doi: 10.1016/s0140-6736(12)61728-0 [published Online First: 2012/12/19]
3. Celli B, MacNee W. Standards for the diagnosis and treatment of patients with COPD: a summary of the ATS/ERS position paper. *The European Respiratory Journal: Official Journal of the European Society for Clinical Respiratory Physiology* 2004;23(6):932 - 46.
4. Maddocks M, Lovell N, Booth S, et al. Palliative care and management of troublesome symptoms for people with chronic obstructive pulmonary disease - The Lancet. 2017 doi: doi:10.1016/S0140-6736(17)32127-X
5. Kelly JL, Bamsey O, Smith C, et al. Health status assessment in routine clinical practice: the chronic obstructive pulmonary disease assessment test score in outpatients. *Respiration* 2012;84(3):193-9. doi: 10.1159/000336549
6. Criner GJ, Cordova F, Sternberg AL, et al. The National Emphysema Treatment Trial (NETT): Part II: Lessons Learned about Lung Volume Reduction Surgery. *Am J Respir Crit Care Med* 2011;184(8):881-93. doi: 10.1164/rccm.201103-0455CI
7. Geddes D, Davies M, Koyama H, et al. Effect of lung-volume-reduction surgery in patients with severe emphysema. *N Engl J Med* 2000;343(4):239-45.

For peer review only

8. National Emphysema Treatment Trial Research Group. A Randomized Trial Comparing Lung-Volume-Reduction Surgery with Medical Therapy for Severe Emphysema. *N Engl J Med* 2003;348(21):2059-73.

9. Ferguson GT, Fernandez E, Zamora MR, et al. Improved exercise performance following lung volume reduction surgery for emphysema. *Am J Respir Crit Care Med* 1998;157(4 Pt 1):1195-203.

10. Clark SJ, Zoumot Z, Bamsey O, et al. Surgical approaches for lung volume reduction in emphysema. *Clinical Medicine* 2014;14(2):122-27. doi: 10.7861/clinmedicine.14-2-122

11. National Institute for Clinical Excellence. Management of chronic obstructive pulmonary disease in adults in primary and secondary care (partial update). This guideline partially updates and replaces NICE clinical guideline 12. *Available from: http://guidanceniceorguk/CG101* 2010

12. Global Initiative for Chronic Obstructive Lung Disease (GOLD) 2017. Global Strategy for the Diagnosis, Management and Prevention of COPD, 2017:http://goldcopd.org.

13. McNulty W, Jordan S, Hopkinson NS. Attitudes and access to lung volume reduction surgery for COPD: a survey by the British Thoracic Society. *BMJ Open Respiratory Research* 2014;1(1) doi: 10.1136/bmjresp-2014-000023

14. Greening NJ, Vaughan P, Oey I, et al. Individualised risk in patients undergoing lung volume reduction surgery: the Glenfield BFG score. *European Respiratory Journal* 2017;49(6) doi: 10.1183/13993003.01766-2016

15. Hopkinson NS, Toma TP, Hansell DM, et al. Effect of Bronchoscopic Lung Volume Reduction on Dynamic Hyperinflation and Exercise in Emphysema. *Am J Respir Crit Care Med* 2005;171(5):453-60.

16. Toma TP, Hopkinson NS, Hillier J, et al. Bronchoscopic volume reduction with valve implants in patients with severe emphysema. *Lancet* 2003;361(9361):931-3. doi: 10.1016/S0140-6736(03)12762-6

For peer review only

17. Snell GI, Holsworth L, Borrill ZL, et al. The potential for bronchoscopic lung volume reduction using bronchial prostheses: a pilot study. *Chest* 2003;124(3):1073-80.

18. Yim AP, Hwong TM, Lee TW, et al. Early results of endoscopic lung volume reduction for emphysema. *The Journal of thoracic and cardiovascular surgery* 2004;127(6):1564-73.

19. Wan IY, Toma TP, Geddes DM, et al. Bronchoscopic lung volume reduction for end-stage emphysema: report on the first 98 patients. *Chest* 2006;129(3):518-26.

20. Venuta F, de Giacomo T, Rendina EA, et al. Bronchoscopic Lung-Volume Reduction With One-Way Valves in Patients With Heterogenous Emphysema. *Ann Thorac Surg* 2005;79(2):411-16.

21. de Oliveira HG, Macedo-Neto AV, John AB, et al. Transbronchoscopic Pulmonary Emphysema Treatment: 1-Month to 24-Month Endoscopic Follow-up. *Chest* 2006;130(1):190-99.

22. Wood DE, McKenna RJ, Jr., Yusen RD, et al. A multicenter trial of an intrabronchial valve for treatment of severe emphysema. *The Journal of thoracic and cardiovascular surgery* 2007;133(1):65-73. doi: 10.1016/j.jtcvs.2006.06.051

23. Hopkinson NS, Kemp SV, Toma TP, et al. Atelectasis and survival after bronchoscopic lung volume reduction for COPD. *European Respiratory Journal* 2010 doi: 10.1183/09031936.00100110

24. Davey C, Zoumot Z, Jordan S, et al. Bronchoscopic lung volume reduction with endobronchial valves for patients with heterogeneous emphysema and intact interlobar fissures (The BeLieVeR-HIFi trial): study design and rationale. *Thorax* 2014 doi: 10.1136/thoraxjnl-2014-205127

25. Davey C, Zoumot Z, Jordan S, et al. Bronchoscopic lung volume reduction with endobronchial valves for patients with heterogeneous emphysema and intact interlobar fissures (the BeLieVeR-HIFi study): a randomised controlled trial. *The Lancet* 2015;386(9998):1066-73. doi: http://dx.doi.org/10.1016/S0140-6736(15)60001-0

For peer review only

26. Herth FJF, Eberhardt R, Gompelmann D, et al. Radiological and clinical outcomes of using Chartis™ to plan endobronchial valve treatment. *European Respiratory Journal* 2013;41(2):302-08. doi: 10.1183/09031936.00015312

27. Kemp SV, Slebos DJ, Kirk A, et al. A Multicenter RCT of Zephyr(R) Endobronchial Valve Treatment in Heterogeneous Emphysema (TRANSFORM). *Am J Respir Crit Care Med* 2017 doi: 10.1164/rccm.201707-1327OC [published Online First: 2017/09/09]

28. Klooster K, ten Hacken NHT, Hartman JE, et al. Endobronchial Valves for Emphysema without Interlobar Collateral Ventilation. *New England Journal of Medicine* 2015;373(24):2325-35. doi: doi:10.1056/NEJMoa1507807

29. Zoumot Z, Davey C, Jordan S, et al. Endobronchial valves for patients with heterogeneous emphysema and without interlobar collateral ventilation: open label treatment following the BeLieVeR-HIFi study. *Thorax* 2017;72(3):277-79. doi: 10.1136/thoraxjnl-2016-208865

30. Williams JE, Green RH, Warrington V, et al. Development of the i-BODE: validation of the incremental shuttle walking test within the BODE index. *Respiratory medicine* 2012;106(3):390-6. doi: 10.1016/j.rmed.2011.09.005 [published Online First: 2011/10/08]

31. Imfeld S, Bloch KE, Weder W, et al. The BODE index after lung volume reduction surgery correlates with survival. *CHEST Journal* 2006;129(4):873-78. doi: 10.1378/chest.129.4.873

32. Rathinam S, Oey I, Steiner M, et al. The role of the emphysema multidisciplinary team in a successful lung volume reduction surgery programmedagger. *European journal of cardio-thoracic surgery : official journal of the European Association for Cardio-thoracic Surgery* 2014;46(6):1021-6; discussion 26. doi: 10.1093/ejcts/ezu129 [published Online First: 2014/04/29]

33. Hakamy A, McKeever TM, Steiner MC, et al. The use of the practice walk test in pulmonary rehabilitation program: National COPD Audit Pulmonary Rehabilitation

For peer review only

Workstream. *International journal of chronic obstructive pulmonary disease* 2017;12:2681-86. doi: 10.2147/copd.s141620 [published Online First: 2017/09/22]

34. Dobbels F, de Jong C, Drost E, et al. The PROactive innovative conceptual framework on physical activity. *European Respiratory Journal* 2014;44(5):1223-33. doi: 10.1183/09031936.00004814
35. Gimeno-Santos E, Frei A, Steurer-Stey C, et al. Determinants and outcomes of physical activity in patients with COPD: a systematic review. *Thorax* 2014;69(8):731-39. doi: 10.1136/thoraxjnl-2013-204763
36. Gimeno-Santos E, Raste Y, Demeyer H, et al. The PROactive instruments to measure physical activity in patients with chronic obstructive pulmonary disease. *European Respiratory Journal* 2015;46(4):988-1000. doi: 10.1183/09031936.00183014
37. Williams JEA, Green RH, Warrington V, et al. Development of the i-BODE: Validation of the incremental shuttle walking test within the BODE index. *Respiratory Medicine* 2012;106(3):390-96. doi: http://dx.doi.org/10.1016/j.rmed.2011.09.005

For peer review only

Figure legends

Figure 1: *schematic* outline of the trial design

For peer review only

	Visit 1<br>Baseline	Visit 2<br>Chartis measurement	Visit 3<br>Admission for procedure	Visit 4<br>One month safety phone call	Visit 5<br>3 months follow up	6 months	9 months	Visit 6<br>1 year<br>Follow up
History, physical exam	x				x			
Informed consent	x							
FFM	x							x
MRC score	x				x			x
CAT score	x				x			x
Full pulmonary function[1]	x				x			x
CT thorax	x				x			
iSWT	x				x			x
Activity monitor given for 7 days	x							x<br>(7 days prior to visit)
Activity monitor collected		x						x
c-PPAC score		x						x
Bronchoscopy with Chartis measurement of collateral ventilation		x						
Randomisation		x						
Treatment intervention:<br>LVRS or BLVR			x					
Health resource utilisation<br>(CSRI questionnaire)					x	x	x	x

Table 1: Summary of trial assessments

[1] Spirometry, gas transfer, plethysmographic lung volumes, capillary blood gases

For peer review only

For peer review only

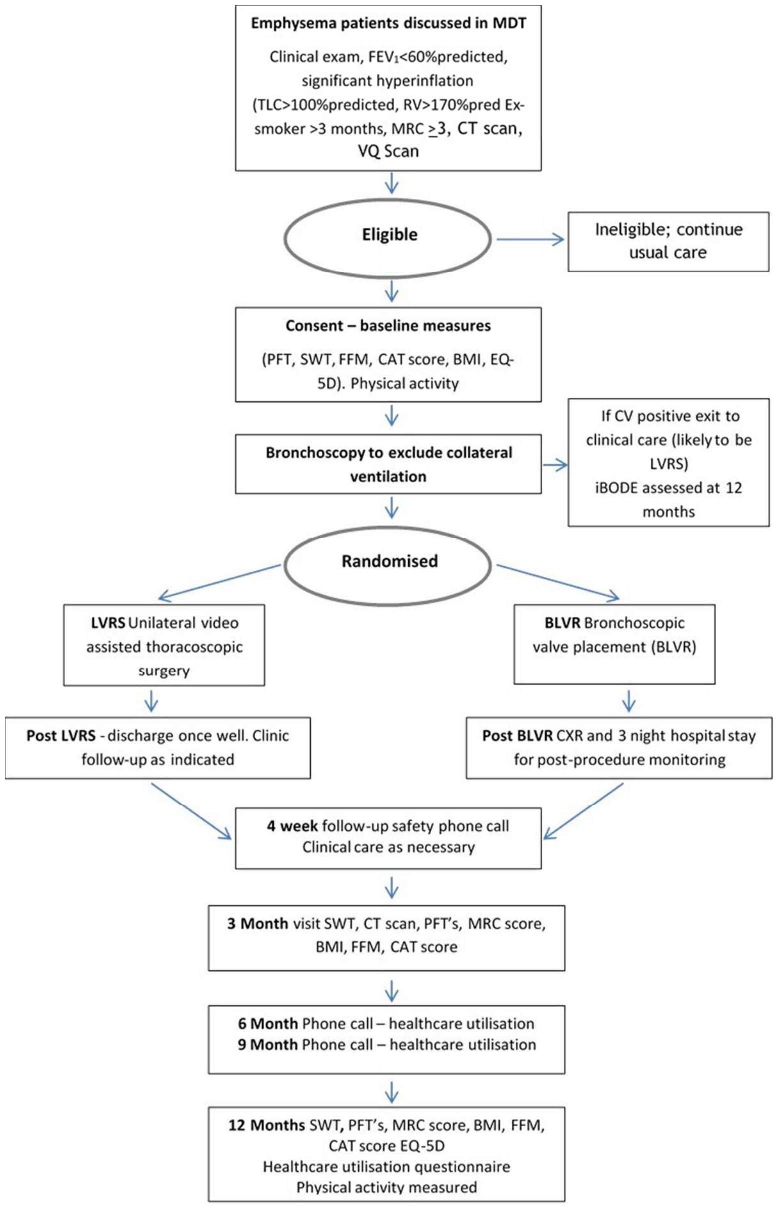

Figure 1

63x99mm (300 x 300 DPI)

SPIRIT
STANDARD PROTOCOL ITEMS: RECOMMENDATIONS FOR INTERVENTIONAL TRIALS

SPIRIT 2013 Checklist: Recommended items to address in a clinical trial protocol and related documents*

Section/item	Item No	Description	Addressed on page number
**Administrative information**			
Title	1	Descriptive title identifying the study design, population, interventions, and, if applicable, trial acronym	**page 1**
Trial registration	2a	Trial identifier and registry name. If not yet registered, name of intended registry	**Page 1: abstract**
	2b	All items from the World Health Organization Trial Registration Data Set	________________
Protocol version	3	Date and version identifier	**page 2: intro**
Funding	4	Sources and types of financial, material, and other support	**Page 13**
Roles and responsibilities	5a	Names, affiliations, and roles of protocol contributors	**Page 12: authors contributions**
	5b	Name and contact information for the trial sponsor	**Page 13**
	5c	Role of study sponsor and funders, if any, in study design; collection, management, analysis, and interpretation of data; writing of the report; and the decision to submit the report for publication, including whether they will have ultimate authority over any of these activities	**Page 13**

For peer review only

For peer review only - http://bmjopen.bmj.com/site/about/guidelines.xhtml

	5d	Composition, roles, and responsibilities of the coordinating centre, steering committee, endpoint adjudication committee, data management team, and other individuals or groups overseeing the trial, if applicable (see Item 21a for data monitoring committee)	**Page 13**
**Introduction**			
Background and rationale	6a	Description of research question and justification for undertaking the trial, including summary of relevant studies (published and unpublished) examining benefits and harms for each intervention	**Page 3/4**
	6b	Explanation for choice of comparators	**Page 3/4**
Objectives	7	Specific objectives or hypotheses	**Page 3/4**
Trial design	8	Description of trial design including type of trial (eg, parallel group, crossover, factorial, single group), allocation ratio, and framework (eg, superiority, equivalence, noninferiority, exploratory)	**Page 3/4**
**Methods: Participants, interventions, and outcomes**			
Study setting	9	Description of study settings (eg, community clinic, academic hospital) and list of countries where data will be collected. Reference to where list of study sites can be obtained	**Page 4/5**
Eligibility criteria	10	Inclusion and exclusion criteria for participants. If applicable, eligibility criteria for study centres and individuals who will perform the interventions (eg, surgeons, psychotherapists)	**Page 4/5**
Interventions	11a	Interventions for each group with sufficient detail to allow replication, including how and when they will be administered	**Page 5**
	11b	Criteria for discontinuing or modifying allocated interventions for a given trial participant (eg, drug dose change in response to harms, participant request, or improving/worsening disease)	**Page 5**
	11c	Strategies to improve adherence to intervention protocols, and any procedures for monitoring adherence (eg, drug tablet return, laboratory tests)	**Page 5**
	11d	Relevant concomitant care and interventions that are permitted or prohibited during the trial	**Page 5**

For peer review only - http://bmjopen.bmj.com/site/about/guidelines.xhtml

Outcomes	12	Primary, secondary, and other outcomes, including the specific measurement variable (eg, systolic blood pressure), analysis metric (eg, change from baseline, final value, time to event), method of aggregation (eg, median, proportion), and time point for each outcome. Explanation of the clinical relevance of chosen efficacy and harm outcomes is strongly recommended	**Page 6**
Participant timeline	13	Time schedule of enrolment, interventions (including any run-ins and washouts), assessments, and visits for participants. A schematic diagram is highly recommended (see Figure)	**Page 7**
Sample size	14	Estimated number of participants needed to achieve study objectives and how it was determined, including clinical and statistical assumptions supporting any sample size calculations	**Page 7**
Recruitment	15	Strategies for achieving adequate participant enrolment to reach target sample size	**Page 7**
**Methods: Assignment of interventions (for controlled trials)**			
Allocation:			
Sequence generation	16a	Method of generating the allocation sequence (e.g., computer-generated random numbers), and list of any factors for stratification. To reduce predictability of a random sequence, details of any planned restriction (eg, blocking) should be provided in a separate document that is unavailable to those who enrol participants or assign interventions	**Page 6**
Allocation concealment mechanism	16b	Mechanism of implementing the allocation sequence (eg, central telephone; sequentially numbered, opaque, sealed envelopes), describing any steps to conceal the sequence until interventions are assigned	**Page 6**
Implementation	16c	Who will generate the allocation sequence, who will enrol participants, and who will assign participants to interventions	**Page 6**
Blinding (masking)	17a	Who will be blinded after assignment to interventions (eg, trial participants, care providers, outcome assessors, data analysts), and how	**Page 6**
	17b	If blinded, circumstances under which unblinding is permissible, and procedure for revealing a participant's allocated intervention during the trial	**Page 6**

Methods: Data collection, management, and analysis

Data collection methods	18a	Plans for assessment and collection of outcome, baseline, and other trial data, including any related processes to promote data quality (eg, duplicate measurements, training of assessors) and a description of study instruments (eg, questionnaires, laboratory tests) along with their reliability and validity, if known. Reference to where data collection forms can be found, if not in the protocol	**Page 8**
	18b	Plans to promote participant retention and complete follow-up, including list of any outcome data to be collected for participants who discontinue or deviate from intervention protocols	**Page 8**
Data management	19	Plans for data entry, coding, security, and storage, including any related processes to promote data quality (eg, double data entry; range checks for data values). Reference to where details of data management procedures can be found, if not in the protocol	**Page 8/9**
Statistical methods	20a	Statistical methods for analysing primary and secondary outcomes. Reference to where other details of the statistical analysis plan can be found, if not in the protocol	**Page 8/9**
	20b	Methods for any additional analyses (eg, subgroup and adjusted analyses)	**Page 8/9**
	20c	Definition of analysis population relating to protocol non-adherence (eg, as randomised analysis), and any statistical methods to handle missing data (eg, multiple imputation)	**Page 8/9**

Methods: Monitoring

Data monitoring	21a	Composition of data monitoring committee (DMC); summary of its role and reporting structure; statement of whether it is independent from the sponsor and competing interests; and reference to where further details about its charter can be found, if not in the protocol. Alternatively, an explanation of why a DMC is not needed	**Page 9**
	21b	Description of any interim analyses and stopping guidelines, including who will have access to these interim results and make the final decision to terminate the trial	**Page 9**
Harms	22	Plans for collecting, assessing, reporting, and managing solicited and spontaneously reported adverse events and other unintended effects of trial interventions or trial conduct	**Page 9**
Auditing	23	Frequency and procedures for auditing trial conduct, if any, and whether the process will be independent from investigators and the sponsor	**Page 9**

For peer review only - http://bmjopen.bmj.com/site/about/guidelines.xhtml

Ethics and dissemination

Research ethics approval	24	Plans for seeking research ethics committee/institutional review board (REC/IRB) approval	**Page 12**
Protocol amendments	25	Plans for communicating important protocol modifications (eg, changes to eligibility criteria, outcomes, analyses) to relevant parties (eg, investigators, REC/IRBs, trial participants, trial registries, journals, regulators)	**Page 12**
Consent or assent	26a	Who will obtain informed consent or assent from potential trial participants or authorised surrogates, and how (see Item 32)	**Page 12**
	26b	Additional consent provisions for collection and use of participant data and biological specimens in ancillary studies, if applicable	**n/a**
Confidentiality	27	How personal information about potential and enrolled participants will be collected, shared, and maintained in order to protect confidentiality before, during, and after the trial	**Page 12**
Declaration of interests	28	Financial and other competing interests for principal investigators for the overall trial and each study site	**Page 12**
Access to data	29	Statement of who will have access to the final trial dataset, and disclosure of contractual agreements that limit such access for investigators	**Page12**
Ancillary and post-trial care	30	Provisions, if any, for ancillary and post-trial care, and for compensation to those who suffer harm from trial participation	**Not included**
Dissemination policy	31a	Plans for investigators and sponsor to communicate trial results to participants, healthcare professionals, the public, and other relevant groups (eg, via publication, reporting in results databases, or other data sharing arrangements), including any publication restrictions	**Page 12**
	31b	Authorship eligibility guidelines and any intended use of professional writers	**Page 12**
	31c	Plans, if any, for granting public access to the full protocol, participant-level dataset, and statistical code	Not included

Appendices

For peer review only - http://bmjopen.bmj.com/site/about/guidelines.xhtml

Informed consent materials	32	Model consent form and other related documentation given to participants and authorised surrogates	**Documents attached to sumission**
Biological specimens	33	Plans for collection, laboratory evaluation, and storage of biological specimens for genetic or molecular analysis in the current trial and for future use in ancillary studies, if applicable	**n/a**

*It is strongly recommended that this checklist be read in conjunction with the SPIRIT 2013 Explanation & Elaboration for important clarification on the items. Amendments to the protocol should be tracked and dated. The SPIRIT checklist is copyrighted by the SPIRIT Group under the Creative Commons "Attribution-NonCommercial-NoDerivs 3.0 Unported" license.

For peer review only

BMJ Open

The CELEB trial: Comparative Effectiveness of Lung volume reduction surgery for Emphysema and Bronchoscopic lung volume reduction with valve placement. A protocol for a randomised controlled trial

Journal:	*BMJ Open*
Manuscript ID	bmjopen-2017-021368.R1
Article Type:	Protocol
Date Submitted by the Author:	04-May-2018

Complete List of Authors:	Buttery, Sara; Royal Brompton and Harefield NHS Foundation Trust,<br>Kemp, Samuel; Royal Brompton and Harefield NHS Foundation Trust<br>Shah, Pallav; Royal Brompton and Harefield NHS Foundation Trust<br>Waller, David; Barts Health NHS Trust<br>Jordan, Simon; Royal Brompton and Harefield NHS Foundation Trust<br>Lee, John; National University of Singapore,<br>Banya, Winston; Royal Brompton and Harefield NHS Foundation Trust<br>Steiner, Michael; Institute for Lung Health,<br>Hopkinson, Nicholas; Royal Brompton Hospital, Respiratory Muscle Lab

**Primary Subject Heading**:	Respiratory medicine
Secondary Subject Heading:	Health economics, Surgery
Keywords:	COPD, Lung Volume Reduction, Thoracic surgery < SURGERY, Emphysema < THORACIC MEDICINE

SCHOLARONE™ Manuscripts

For peer review only

The CELEB trial: Comparative Effectiveness of Lung volume reduction surgery for Emphysema and Bronchoscopic lung volume reduction with valve placement.

A protocol for a randomised controlled trial

[1]Sara Buttery, [1]Samuel V. Kemp, [1]Pallav L. Shah [3] David Waller, [1]Simon Jordan, [4]J L. Lee, [1]W. Banya, [2]Michael Steiner, *[1]Nicholas S. Hopkinson

[1]NIHR Respiratory Disease, Biomedical Research Unit at the Royal Brompton and Harefield NHS Foundation Trust and Imperial College London, London, UK

[2] Leicester Respiratory Biomedical Research Unit, University Hospitals of Leicester NHS Foundation Trust, Leicester, UK

[3] Barts Health NHS Trust London, London, UK

[4] National University of Singapore, Singapore, SG119260

Corresponding author: S.Buttery

Royal Brompton Hospital

Fulham Road

London

SW3 6HP

Email: s.buttery@rbht.nhs.uk

Telephone: 0207 351 8029

Fax: 0207 351 8939

For peer review only

For peer review only - http://bmjopen.bmj.com/site/about/guidelines.xhtml

Abstract:

Introduction: Although lung volume reduction surgery and bronchoscopic lung volume reduction with endobronchial valves have both been shown to improve lung function, exercise capacity and quality of life in appropriately selected patients with emphysema, there are no direct comparison data between the two procedures to inform clinical decision making.

Methods and Analysis: We describe the protocol of the CELEB study (**ISRCTN**19684749), a randomised controlled trial which will compare outcomes at 1 year between the two procedures, using a composite disease severity measure, the iBODE score, which includes body mass index, lung function, breathlessness and exercise capacity.

Ethics and Dissemination: Ethical approval to conduct the study has been obtained from the Fulham Research Ethics Committee, London (16/LO/0286). The outcome of this trial will provide information to guide treatment choices in this population and will be presented at national and international meetings and published in peer-reviewed journals. We will also disseminate the main results to all participants in a letter.

Strengths and Limitations

- This is a randomised controlled trial
- There is a strict patient selection criterion for this trial based on previous research.
- Currently there is equipoise between the two interventions being compared.
- Due to the nature of the interventions it is not possible to double blind the trial.
- It may be difficult to obtain longer term outcomes as patients may cross over after the 12 month period of the trial.

For peer review only

Introduction:

Chronic obstructive pulmonary disease (COPD) is a common and disabling condition which is now the third largest cause of death worldwide[1]. Breathlessness, exercise limitation and mortality in COPD are all associated with increased lung volumes occurring due to airflow obstruction and increased lung complicance[2]. The condition is progressive and despite optimum care including smoking cessation, pharmacotherapy and pulmonary rehabilitation, many patients remain breathless and limited in everyday activities[3 4]. Surgical and bronchoscopic approaches to lung volume reduction are available which can bring substantial benefits in appropriately selected individuals, though both are also associated with some risk.

Lung volume reduction surgery (LVRS) involves removing the worst affected area of emphasematous lung, which means that the remaining healthier and less compliant lung can function more effectively, with the respiratory muscles working at less of a mechanical discadvantages[5]. LVRS has been shown to improve survival, exercise capacity and quality of life in appropriately selected patients with heterogeneous emphysema and poor exercise capacity[5-9]. and is therefore recommended in national and international guidelines for the management of COPD[1 10 11]. However, uptake has been limited, due in part to exaggerated concerns about surgical morbidity and mortality[12]. In modern clinical practice morbidity and mortality from the procedure is substantially lower[9 13] than was the case in trials conducted around the turn of the century[5 6].

An alterative approach to resecting the target area of lung is to place endobronchial valves occluding the airways supplying a particular lobe. This form of bronchoscopic lung volume reduction (BLVR) can produce lobar atelectasis, and is intended to achieve similar benefits to LVRS but with less morbidity[14-24]. The approach is only effective in the absence of

For peer review only - http://bmjopen.bmj.com/site/about/guidelines.xhtml

interlobar collateral ventilation (CV)[23 25]. If this is present air can enter the target lobe from an adjacent lobe and atelectasis does not occur. In patients with a heterogenous pattern of emphysema and intact interlobar fissures, valve placement can produce significant improvements in lung function, exercise capacity and health status[23 26-28]. Yet complications do occur, in particular pneumothorax which occurs in up to 30% of cases[28] and can on occasion be fatal, as well as exacerbation-like events. Valve expectoration or misplacement can necessitate additional procedures.

Patients with heterogeneous emphysema and intact interlobar fissures, may benefit from either BLVR or LVRS, but there are no direct comparison data on the relative value of the two procedures to guide clinical decision making.

The CELEB study is a multi-centre, randomised – controlled parallel group trial that opened for recruitment in October 2016. Patients, who are considered to be suitable candidates for both forms of targeted lung reduction therapy, will be randomised to either BLVR or LVRS. The primary aim of CELEB study is to determine wether LVRS procedures a greater health benefit at one year than BLVR. This will be determined using change in the iBODE index, a composite meansure of COPD severity (made up of BMI, lung function, breathlessness and exercise capacity)[29]. Change in BODE score has been found to be a good predictor of survival in LVRS patients[30]. Health resource ultisation will also be compared between treatment arms. Clinical efficacy will be assessed over a 12 month period. The study is set up as a superiority trial and will be conducted, analysed and reported according to the Consolidation Standards of Reporting Trials (CONSROT) statement for randomised controlled trials. This paper will outline the protocol for the CELEB study (version 2.0, 19th February 2016).

For peer review only - http://bmjopen.bmj.com/site/about/guidelines.xhtml

Methods and Analysis:

Participants: Patients will be recruited from outpatient clinics at UK hospital sites which have an established multi-disciplinary team (MDT) meeting dedicated to identifying suitable candidates for LVR[31]. Patients will be eligible for the study if they fit the following criteria; significant airflow obstruction (FEV_1<60% predicted), limiting breathlessness (MRC dyspnoea score > 3), significant hyperinflation (TLC>100% predicted, RV>170% predicted) and are considered, using CT and perfusion scan, to have a heterogeneous emphysema pattern with intact interlobar fissures (>90%). They must also not have smoked for at least 3 months. Patients fitting these criteria will be identified from outpatient clinics and put forward for discussion at the MDT meeting. Patients will be excluded if they have pulmonary fibrosis or any other major co-morbidity that could affect survival or mean that LVR procedures are unlikely to be effective. Patients with PaO_2<7.0kPa and/or $PaCO_2$>7.0kPa will also be excluded. The MDT will make a decision on whether a patient is suitable for both interventions and if so, that there is equipoise between the treatments options. It is only after this is agreed that the screening visit will be arranged and consent taken.

Interventions: LVRS will be carried out by a thoracic surgeon under general anaesthetic using a unilateral VATS approach. The precise operation will be at the discretion of the surgeon, intended to remove the most emphysematous area of lung, leaving the best quality lung behind and minimising air leaks and other complications. Patients will initially go to the High Dependency Unit (HDU) post operatively and post-operative management will include attention to wound discomfort, management of chest drains and prompt mobilisation. Patient management, discharge and initial follow up will be determined by their clinical team.

For peer review only - http://bmjopen.bmj.com/site/about/guidelines.xhtml

BLVR will be performed via bronchoscopy by an operator experienced in placing EBV. The procedure will be performed under conscious sedation or general anaesthetic if necessary, with endobronchial valves placed to occlude the target lobe. A chest x-ray will be performed one hour post procedure and participants will spend three nights post-procedure as an in-patient routinely in case a pneumothorax occurs. They will be discharged with written advice about the signs of pneumothorax and what to do if these occur. Procedures including valve adjustment and replacement will be permitted to ensure treatment is optimised.

Participants will be followed up in clinic routinely about three months after discharge. Other clinic attendances will depend on clinical need with further investigations and procedures determined by their responsible clinician. Patients will receive a phone call from the study team at one month and at 6 and 9 months. There will be a final clinic visit at 12 months. A summary of the trial assessment is shown in **Table 1.**

Outcomes: The Primary outcome will be the change in iBODE score[32] at 12 months from baseline. The iBODE incorporates the incremental shuttle walk test, BMI, FEV_1 and the MRC dyspnoea score. Possible scores range from 0 to 10 with 10 with increasing scores associated with worsening mortality. The primary endpoint measures will be performed by staff blind to treatment allocation and patients will be asked not to reveal this in order to reduce bias.

Secondary outcomes will be assessed at baseline, 3 months and 12 months. Changes in respiratory related health status (COPD assessment test (CAT)), health related quality of life (HRQOL) using the EQ-5D-5L residual volume (RV) and fat free mass (FFM) will be evaluated. Physical activity will be measured using the Proactive c-PPAC score.

The basis for the sample size calculation is the paper by Imfeld *et al* which addressed the response to LVRS[30]. This compared the improvement in BODE score at three months post LVRS between survivors and non-survivors at 5 years. Long term survivors had an improvement of 3.9 points at three months, the non–survivors, had an improvement of 2.4

For peer review only - http://bmjopen.bmj.com/site/about/guidelines.xhtml

points (*i.e.* a difference of 1.5 points). This difference is likely therefore to represent a clinically important difference between outcomes in the two LVR arms, being of sufficient magnitude to influence treatment decisions and is therefore a meaningful basis on which to power the study.

The study is powered on the basis of an average response to LVRS that will be the same as in the Imfeld paper (*i.e.* a fall of 3.1) and for BLVR that the improvement will be 1.6 (1.5 points less benefit). Assuming an SD of 1.8 for the change in BODE score and taking a 5% significance level and 90% statistical power, 34 patients would be required in each treatment arm. Allowing for 10% drop-outs we will enrol 76 individuals.

Assignment of interventions: All participants will undergo a fibreoptic bronchoscopy to allow for assessment of the presence of collateral ventilation using the Chartis^{TM} system[25] This uses a balloon catheter, inflated in the target airway, with a flow sensor and a distal pressure meter. Pressure swings should continue with inspiration and expiration, but flow will diminish and then cease as the target lobe empties if there is no collateral ventilation (CV –ve). If collateral ventilation is present, flow will continue (CV +ve). Subsequent participation in the trial depends on this. As the absence of collateral ventilation in the target lobe is an inclusion criteria patients with collateral ventilation are deemed to be screen failures and cannot proceed to randomisation (as they would not benefit from valve placement so there is no longer equipoise). However they will still be suitable for a surgical approach and may go on to "open label" LVRS. We will endeavour to follow these patients up at 12 months and their iBODE score will be evaluated. In some cases CV cannot be assessed either because there is continuing low flow or an abrupt cessation in the flow trace suggesting obstruction. It may be possible to work round this by measuring the presence or absence of CV in the adjacent lobe (*i.e.* the reciprocal position). Where CV is truly indeterminate, patients will be withdrawn from the study (*i.e.* treated as CV positive).

For peer review only - http://bmjopen.bmj.com/site/about/guidelines.xhtml

If the person has a CV negative lobar target for treatment they will be immediately allocated randomly to one of the treatment arms, using an online system. Randomisation will be on a 1:1 basis based on a computer generated random sequence with random block sizes generated by Sealed Envelope ltd (London, UK). This will include stratification by site and by iBODE score to ensure matched numbers in both arms with an iBODE score of >7.

Patients, the study co-ordinator and those providing clinical care will not be blind to treatment allocation. The assessors recording the components of the iBODE score will be blinded to reduce the potential for bias. Therefore no un-blinding process will be necessary.

Data collection, management and analysis:

At baseline a full medical history including exacerbations, hospital admissions and drug history will be taken. Pulmonary Function Tests (spirometry, plethysmography and gas transfer measurements) will be carried out according to the American Thoracic Society/European Respiratory Society recommendations, together with arterial blood gas analysis. Additional measures will include Medical Research Council dyspnoea score, health status (CAT), HRQOL (EQ-5D-5L) and fat free mass using bioimpedance. Two incremental shuttle walk tests (ISWT) will be carried out due to the learning effect seen following a test walk[32].

Phyiscal activity will be assessed using the PROactive Consortium patient reported outcome tool for physical activity in COPD[33-35]. Participants wear a Dynaport Movemonitor accelerometer for one week as a measure of physical activity at the end of this they complete the c-PACC physical activity questionnaire which asks about the amount and difficulty of physical activity during the period covered by the monitor.

For peer review only - http://bmjopen.bmj.com/site/about/guidelines.xhtml

A cost-utility analysis will be carried out from a National Health Service (NHS) perspective using data from the trial. The relative value of the two approaches will be based on procedural cost, length of stay, days in hospital subsequently expressed as a cost per quality-adjusted life year (QALY) together with data gathered using the Client Socio-Demographic and Service Receipt Inventory (CSRI) which asks participants about their use of health and social services over the last three months. This will be completed at 3, 6, 9 and 12 months. They will be asked to report the frequency and intensity of their service use. Cost incurred in both arms may include (i) medical and surgical services received as an in-patient (ii) health care visits in relation to COPD (iii) COPD related medication use. Total cost of individuals will be estimated by multiplying resources use with national tariff used in the NHS. QALY will be estimated using EQ-5D-5L questionnaire. The five attributes of this questionnaire (mobility, self-care, usual activities, pain/discomfort/ anxiety/depression) will be summarised into a single UK-derived preference-based utility score.

Figure 1 provides a summary of the trial design.

Figure 1

Outcome data analysis will be by intention to treat. Patients with missing data will be assumed to have had zero change from baseline. Unpaired t-tests will be used to compare mean response across the 2 groups: control and intervention. Mixed linear model procedure to evaluate the effects of predefined covariables on dependent outcome variables.

For peer review only - http://bmjopen.bmj.com/site/about/guidelines.xhtml

Total costs will be calculated as the sum-product of resource use and unit cost for each patient. Incremental cost-effectiveness ratios (ICER) will be obtained by calculating the incremental costs divided by the incremental effects for the LVRS vs the BLVR groups`. Multivariate regression analysis will be used to adjust for baseline differences in health status between the trial groups. Uncertainty in the incremental cost-effectiveness ratio (ICER) will be addressed using bootstrapping and the estimation of cost-effectiveness acceptability curves. Estimates of ICERs will be compared with the £20,000 to £30,000 per QALY threshold of cost-effectiveness recommended by the National Institute for Health and Care Excellence (NICE). No interim analyses are planned and the trial has no formal stopping guidelines.

Patient and public involvement

Prior to commencement of the trial we held two focus groups of patients with COPD who have undergone LVRS and/or BLVR. This allowed us to capture qualitative information about issues of importance to this group of patients beyond those captured by generic or disease specific health status tools. Participants will also be invited to help with reviewing dissemination materials.

For peer review only

For peer review only - http://bmjopen.bmj.com/site/about/guidelines.xhtml

.

Ethics and dissemination: Ethical approval to conduct the study has been obtained from the Fulham Research Ethics Committee, London (16/LO/0286)

Trial procedures will only begin following a clinical decision by the MDT that a patient is eligible for BLVR or LVRS. Patients will then receive verbal and written information regarding the study and informed written consent will be taken at the screening visit prior to any measurements being taken. A trial steering committee will provide overall supervision of the trial and ensures that it is being conducted in accordance with the principles of Good Clinical Practice (GCP) standards.

Contributorship Statements: This protocol was written by the Chief Investigator (CI) Nicolas Hopkinson (NSH) in accordance with the Sponsor's guidance for writing non-CTIMP protocols. NSH and the Research Office discussed and agreed this study protocol. All other applicants SB, SVK, PLS, DW, SJ, JLL, WB, MS were involved in trial design and revisions of the final protocol and will be involved in the production of the final manuscript upon trial completion. All investigators (NSH, SB, SVK, PLS, DW, SJ, MS) agreed to perform the investigations outlined in the study protocol and to abide by this protocol except in the case of medical emergency that will be notified to the Research Office. All authors have agreed upon the final manuscript for this submission.

We would also like to thank the patient advisors that attended the focus groups and contributed to the design of this study.

For peer review only - http://bmjopen.bmj.com/site/about/guidelines.xhtml

Funding Statement: The study is funded by a grant to Royal Brompton and Harefield NHS Foundation Trust, from the NIHR, through the Research for Patient Benefit scheme (PB-PG-1014-35051). Imperial College, London will support the reporting of this manuscript.

Competing Interests Statement: No support from any external organisation or financial relationships with any organisations that might have an interest in the submitted work has been sought for this study. Royal Brompton has received reimbursement of clinical trial expenses from PneumRx, PulmonX, Olympus, Uptake Medical, Holaira, and Creo Medical. The sponsor will not be involved in the conduct of the trial or influence any decision to publish.

Trial sponsor representative

Mr Patrik Pettersson

Royal Brompton and Harefield NHS Foundation Trust (RB&HFT)

Royal Brompton Hospital

Research office

Chelsea Wing, Level 2

Sydney Street

London , SW3 6NP

For peer review only

References

1. Lozano R, Naghavi M, Foreman K, et al. Global and regional mortality from 235 causes of death for 20 age groups in 1990 and 2010: a systematic analysis for the Global Burden of Disease Study 2010. *Lancet (London, England)* 2012;380(9859):2095-128. doi: 10.1016/s0140-6736(12)61728-0 [published Online First: 2012/12/19]

2. Celli BR, MacNee W. Standards for the diagnosis and treatment of patients with COPD: a summary of the ATS/ERS position paper. *The European respiratory journal* 2004;23(6):932-46. [published Online First: 2004/06/29]

3. Maddocks M, Lovell N, Booth S, et al. Palliative care and management of troublesome symptoms for people with chronic obstructive pulmonary disease. *Lancet (London, England)* 2017;390(10098):988-1002. doi: 10.1016/s0140-6736(17)32127-x [published Online First: 2017/09/06]

4. Kelly JL, Bamsey O, Smith C, et al. Health status assessment in routine clinical practice: the chronic obstructive pulmonary disease assessment test score in outpatients. *Respiration; international review of thoracic diseases* 2012;84(3):193-9. doi: 10.1159/000336549 [published Online First: 2012/03/24]

5. Criner GJ, Cordova F, Sternberg AL, et al. The National Emphysema Treatment Trial (NETT) Part II: Lessons learned about lung volume reduction surgery. *American journal of respiratory and critical care medicine* 2011;184(8):881-93. doi: 10.1164/rccm.201103-0455CI [published Online First: 2011/07/02]

6. Geddes D, Davies M, Koyama H, et al. Effect of lung-volume-reduction surgery in patients with severe emphysema. *The New England journal of medicine* 2000;343(4):239-45. doi: 10.1056/nejm200007273430402 [published Online First: 2000/07/27]

7. Fishman A, Martinez F, Naunheim K, et al. A randomized trial comparing lung-volume-reduction surgery with medical therapy for severe emphysema. *The New England journal of medicine* 2003;348(21):2059-73. doi: 10.1056/NEJMoa030287 [published Online First: 2003/05/22]

8. Ferguson GT, Fernandez E, Zamora MR, et al. Improved exercise performance following lung volume reduction surgery for emphysema. *American journal of respiratory and critical care medicine* 1998;157(4 Pt 1):1195-203. doi: 10.1164/ajrccm.157.4.9705008 [published Online First: 1998/05/01]

For peer review only - http://bmjopen.bmj.com/site/about/guidelines.xhtml

9. Clark SJ, Zoumot Z, Bamsey O, et al. Surgical approaches for lung volume reduction in emphysema. *Clinical medicine (London, England)* 2014;14(2):122-7. doi: 10.7861/clinmedicine.14-2-122 [published Online First: 2014/04/10]

10. Chronic obstructive pulmonary disease in adults | Guidance and guidelines | NICE: NICE; 2018 [Available from: https://www.nice.org.uk/guidance/qs10.

11. GOLD 2017 Global Strategy for the Diagnosis, Management and Prevention of COPD - Global Initiative for Chronic Obstructive Lung Disease - GOLD 2018 [Available from: http://goldcopd.org/gold-2017-global-strategy-diagnosis-management-prevention-copd/.

12. McNulty W, Jordan S, Hopkinson NS. Attitudes and access to lung volume reduction surgery for COPD: a survey by the British Thoracic Society. *BMJ open respiratory research* 2014;1(1):e000023. doi: 10.1136/bmjresp-2014-000023 [published Online First: 2014/12/06]

13. Greening NJ, Vaughan P, Oey I, et al. Individualised risk in patients undergoing lung volume reduction surgery: the Glenfield BFG score. *The European respiratory journal* 2017;49(6) doi: 10.1183/13993003.01766-2016 [published Online First: 2017/06/03]

14. Yim AP, Hwong TM, Lee TW, et al. Early results of endoscopic lung volume reduction for emphysema. *The Journal of thoracic and cardiovascular surgery* 2004;127(6):1564-73. doi: 10.1016/j.jtcvs.2003.10.005 [published Online First: 2004/06/03]

15. Wood DE, McKenna RJ, Jr., Yusen RD, et al. A multicenter trial of an intrabronchial valve for treatment of severe emphysema. *The Journal of thoracic and cardiovascular surgery* 2007;133(1):65-73. doi: 10.1016/j.jtcvs.2006.06.051 [published Online First: 2007/01/03]

16. Wan IY, Toma TP, Geddes DM, et al. Bronchoscopic lung volume reduction for end-stage emphysema: report on the first 98 patients. *Chest* 2006;129(3):518-26. doi: 10.1378/chest.129.3.518 [published Online First: 2006/03/16]

17. Venuta F, de Giacomo T, Rendina EA, et al. Bronchoscopic lung-volume reduction with one-way valves in patients with heterogenous emphysema. *The Annals of thoracic*

For peer review only - http://bmjopen.bmj.com/site/about/guidelines.xhtml

surgery 2005;79(2):411-6; discussion 16-7. doi: 10.1016/j.athoracsur.2004.07.048 [published Online First: 2005/02/01]

18. Toma TP, Hopkinson NS, Hillier J, et al. Bronchoscopic volume reduction with valve implants in patients with severe emphysema. *Lancet (London, England)* 2003;361(9361):931-3. doi: 10.1016/s0140-6736(03)12762-6 [published Online First: 2003/03/22]

19. Snell GI, Holsworth L, Borrill ZL, et al. The potential for bronchoscopic lung volume reduction using bronchial prostheses: a pilot study. *Chest* 2003;124(3):1073-80. [published Online First: 2003/09/13]

20. Hopkinson NS, Toma TP, Hansell DM, et al. Effect of bronchoscopic lung volume reduction on dynamic hyperinflation and exercise in emphysema. *American journal of respiratory and critical care medicine* 2005;171(5):453-60. doi: 10.1164/rccm.200407-961OC [published Online First: 2004/12/08]

21. Hopkinson NS, Kemp SV, Toma TP, et al. Atelectasis and survival after bronchoscopic lung volume reduction for COPD. *The European respiratory journal* 2011;37(6):1346-51. doi: 10.1183/09031936.00100110 [published Online First: 2010/10/16]

22. de Oliveira HG, Macedo-Neto AV, John AB, et al. Transbronchoscopic pulmonary emphysema treatment: 1-month to 24-month endoscopic follow-up. *Chest* 2006;130(1):190-9. doi: 10.1378/chest.130.1.190 [published Online First: 2006/07/15]

23. Davey C, Zoumot Z, Jordan S, et al. Bronchoscopic lung volume reduction with endobronchial valves for patients with heterogeneous emphysema and intact interlobar fissures (the BeLieVeR-HIFi study): a randomised controlled trial. *Lancet (London, England)* 2015;386(9998):1066-73. doi: 10.1016/s0140-6736(15)60001-0 [published Online First: 2015/06/28]

24. Davey C, Zoumot Z, Jordan S, et al. Bronchoscopic lung volume reduction with endobronchial valves for patients with heterogeneous emphysema and intact interlobar fissures (the BeLieVeR-HIFi trial): study design and rationale. *Thorax* 2015;70(3):288-90. doi: 10.1136/thoraxjnl-2014-205127 [published Online First: 2014/03/26]

For peer review only - http://bmjopen.bmj.com/site/about/guidelines.xhtml

25. Herth FJ, Eberhardt R, Gompelmann D, et al. Radiological and clinical outcomes of using Chartis to plan endobronchial valve treatment. *The European respiratory journal* 2013;41(2):302-8. doi: 10.1183/09031936.00015312 [published Online First: 2012/05/05]

26. Zoumot Z, Davey C, Jordan S, et al. Endobronchial valves for patients with heterogeneous emphysema and without interlobar collateral ventilation: open label treatment following the BeLieVeR-HIFi study. Thorax. England: Published by the BMJ Publishing Group Limited. For permission to use (where not already granted under a licence) please go to http://www.bmj.com/company/products-services/rights-and-licensing/. 2017:277-79.

27. Klooster K, ten Hacken NH, Hartman JE, et al. Endobronchial Valves for Emphysema without Interlobar Collateral Ventilation. *The New England journal of medicine* 2015;373(24):2325-35. doi: 10.1056/NEJMoa1507807 [published Online First: 2015/12/10]

28. Kemp SV, Slebos DJ, Kirk A, et al. A Multicenter Randomized Controlled Trial of Zephyr Endobronchial Valve Treatment in Heterogeneous Emphysema (TRANSFORM). *American journal of respiratory and critical care medicine* 2017;196(12):1535-43. doi: 10.1164/rccm.201707-1327OC [published Online First: 2017/09/09]

29. Williams JE, Green RH, Warrington V, et al. Development of the i-BODE: validation of the incremental shuttle walking test within the BODE index. *Respiratory medicine* 2012;106(3):390-6. doi: 10.1016/j.rmed.2011.09.005 [published Online First: 2011/10/08]

30. Imfeld S, Bloch KE, Weder W, et al. The BODE index after lung volume reduction surgery correlates with survival. *Chest* 2006;129(4):873-8. doi: 10.1378/chest.129.4.873 [published Online First: 2006/04/13]

31. Rathinam S, Oey I, Steiner M, et al. The role of the emphysema multidisciplinary team in a successful lung volume reduction surgery programmedagger. *European journal of cardio-thoracic surgery : official journal of the European Association for Cardio-thoracic Surgery* 2014;46(6):1021-6; discussion 26. doi: 10.1093/ejcts/ezu129 [published Online First: 2014/04/29]

For peer review only - http://bmjopen.bmj.com/site/about/guidelines.xhtml

32. Hakamy A, McKeever TM, Steiner MC, et al. The use of the practice walk test in pulmonary rehabilitation program: National COPD Audit Pulmonary Rehabilitation Workstream. *International journal of chronic obstructive pulmonary disease* 2017;12:2681-86. doi: 10.2147/copd.s141620 [published Online First: 2017/09/22]

33. Dobbels F, de Jong C, Drost E, *et al*. The PROactive innovative conseptual framework on physical activity. *European Respiratory Journal* 2014;44(5):1223-33. doi: 10.1136/thoraxjnl-2013-204763

34. Gimeno-Santos E, Raste Y, Demeyer H, et al. The PROactive instruments to measure physical activity in patients with chronic obstructive pulmonary disease. *The European respiratory journal* 2015;46(4):988-1000. doi: 10.1183/09031936.00183014 [published Online First: 2015/05/30]

35. Gimeno-Santos E, Frei A, Steurer-Stey C, et al. Determinants and outcomes of physical activity in patients with COPD: a systematic review. *Thorax* 2014;69(8):731-9. doi: 10.1136/thoraxjnl-2013-204763 [published Online First: 2014/02/22]

For peer review only

Figure Legends

Figure 1: *schematic* outline of the trial design

For peer review only

For peer review only - http://bmjopen.bmj.com/site/about/guidelines.xhtml

	Visit 1<br>Baseline	Visit 2<br>Chartis measurement	Visit 3<br>Admission for procedure	Visit 4<br>One month safety phone call	Visit 5<br>3 months follow up	6 months	9 months	Visit 6<br>1 year<br>Follow up
History, physical exam	x				x			
Informed consent	x							
FFM	x							x
MRC score	x				x			x
CAT score	x				x			x
Full pulmonary function[1]	x				x			x
CT thorax	x				x			
iSWT	x				x			x
Activity monitor given for 7 days	x							x<br>(7 days prior to visit)
Activity monitor collected		x						x
c-PPAC score		x						x
Bronchoscopy with Chartis measurement of collateral ventilation		x						
Randomisation		x						
Treatment intervention:<br>LVRS or BLVR			x					
Health resource utilisation<br>(CSRI questionnaire)					x	x	x	x

Table 1: Summary of trial assessments

[1] Spirometry, gas transfer, plethysmographic lung volumes, capillary blood gases

For peer review only

For peer review only

For peer review only - http://bmjopen.bmj.com/site/about/guidelines.xhtml

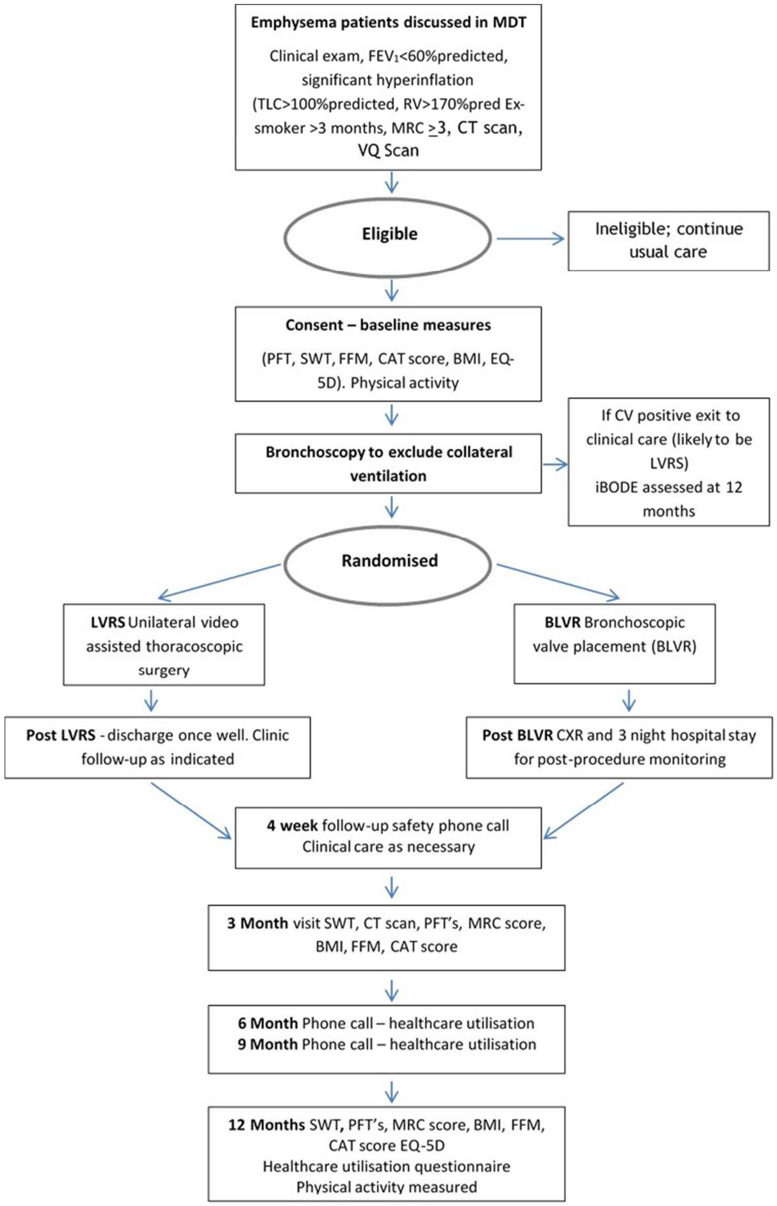

Figure 1

63x99mm (300 x 300 DPI)

STANDARD PROTOCOL ITEMS: RECOMMENDATIONS FOR INTERVENTIONAL TRIALS

SPIRIT 2013 Checklist: Recommended items to address in a clinical trial protocol and related documents*

Section/item	Item No	Description	Addressed on page number
**Administrative information**			
Title	1	Descriptive title identifying the study design, population, interventions, and, if applicable, trial acronym	**page 1**
Trial registration	2a	Trial identifier and registry name. If not yet registered, name of intended registry	**Page 1: abstract**
	2b	All items from the World Health Organization Trial Registration Data Set	______________
Protocol version	3	Date and version identifier	**page 2: intro**
Funding	4	Sources and types of financial, material, and other support	**Page 13**
Roles and responsibilities	5a	Names, affiliations, and roles of protocol contributors	**Page 12: authors contributions**
	5b	Name and contact information for the trial sponsor	**Page 13**
	5c	Role of study sponsor and funders, if any, in study design; collection, management, analysis, and interpretation of data; writing of the report; and the decision to submit the report for publication, including whether they will have ultimate authority over any of these activities	**Page 13**

For peer review only

For peer review only - http://bmjopen.bmj.com/site/about/guidelines.xhtml

	5d	Composition, roles, and responsibilities of the coordinating centre, steering committee, endpoint adjudication committee, data management team, and other individuals or groups overseeing the trial, if applicable (see Item 21a for data monitoring committee)	**Page 13**
**Introduction**			
Background and rationale	6a	Description of research question and justification for undertaking the trial, including summary of relevant studies (published and unpublished) examining benefits and harms for each intervention	**Page 3/4**
	6b	Explanation for choice of comparators	**Page 3/4**
Objectives	7	Specific objectives or hypotheses	**Page 3/4**
Trial design	8	Description of trial design including type of trial (eg, parallel group, crossover, factorial, single group), allocation ratio, and framework (eg, superiority, equivalence, noninferiority, exploratory)	**Page 3/4**
**Methods: Participants, interventions, and outcomes**			
Study setting	9	Description of study settings (eg, community clinic, academic hospital) and list of countries where data will be collected. Reference to where list of study sites can be obtained	**Page 4/5**
Eligibility criteria	10	Inclusion and exclusion criteria for participants. If applicable, eligibility criteria for study centres and individuals who will perform the interventions (eg, surgeons, psychotherapists)	**Page 4/5**
Interventions	11a	Interventions for each group with sufficient detail to allow replication, including how and when they will be administered	**Page 5**
	11b	Criteria for discontinuing or modifying allocated interventions for a given trial participant (eg, drug dose change in response to harms, participant request, or improving/worsening disease)	**Page 5**
	11c	Strategies to improve adherence to intervention protocols, and any procedures for monitoring adherence (eg, drug tablet return, laboratory tests)	**Page 5**
	11d	Relevant concomitant care and interventions that are permitted or prohibited during the trial	**Page 5**

For peer review only - http://bmjopen.bmj.com/site/about/guidelines.xhtml

Outcomes	12	Primary, secondary, and other outcomes, including the specific measurement variable (eg, systolic blood pressure), analysis metric (eg, change from baseline, final value, time to event), method of aggregation (eg, median, proportion), and time point for each outcome. Explanation of the clinical relevance of chosen efficacy and harm outcomes is strongly recommended	**Page 6**
Participant timeline	13	Time schedule of enrolment, interventions (including any run-ins and washouts), assessments, and visits for participants. A schematic diagram is highly recommended (see Figure)	**Page 7**
Sample size	14	Estimated number of participants needed to achieve study objectives and how it was determined, including clinical and statistical assumptions supporting any sample size calculations	**Page 7**
Recruitment	15	Strategies for achieving adequate participant enrolment to reach target sample size	**Page 7**
**Methods: Assignment of interventions (for controlled trials)**			
Allocation:			
Sequence generation	16a	Method of generating the allocation sequence (e.g., computer-generated random numbers), and list of any factors for stratification. To reduce predictability of a random sequence, details of any planned restriction (eg, blocking) should be provided in a separate document that is unavailable to those who enrol participants or assign interventions	**Page 6**
Allocation concealment mechanism	16b	Mechanism of implementing the allocation sequence (eg, central telephone; sequentially numbered, opaque, sealed envelopes), describing any steps to conceal the sequence until interventions are assigned	**Page 6**
Implementation	16c	Who will generate the allocation sequence, who will enrol participants, and who will assign participants to interventions	**Page 6**
Blinding (masking)	17a	Who will be blinded after assignment to interventions (eg, trial participants, care providers, outcome assessors, data analysts), and how	**Page 6**
	17b	If blinded, circumstances under which unblinding is permissible, and procedure for revealing a participant's allocated intervention during the trial	**Page 6**

For peer review only - http://bmjopen.bmj.com/site/about/guidelines.xhtml

Methods: Data collection, management, and analysis

Data collection methods	18a	Plans for assessment and collection of outcome, baseline, and other trial data, including any related processes to promote data quality (eg, duplicate measurements, training of assessors) and a description of study instruments (eg, questionnaires, laboratory tests) along with their reliability and validity, if known. Reference to where data collection forms can be found, if not in the protocol	**Page 8**
	18b	Plans to promote participant retention and complete follow-up, including list of any outcome data to be collected for participants who discontinue or deviate from intervention protocols	**Page 8**
Data management	19	Plans for data entry, coding, security, and storage, including any related processes to promote data quality (eg, double data entry; range checks for data values). Reference to where details of data management procedures can be found, if not in the protocol	**Page 8/9**
Statistical methods	20a	Statistical methods for analysing primary and secondary outcomes. Reference to where other details of the statistical analysis plan can be found, if not in the protocol	**Page 8/9**
	20b	Methods for any additional analyses (eg, subgroup and adjusted analyses)	**Page 8/9**
	20c	Definition of analysis population relating to protocol non-adherence (eg, as randomised analysis), and any statistical methods to handle missing data (eg, multiple imputation)	**Page 8/9**

Methods: Monitoring

Data monitoring	21a	Composition of data monitoring committee (DMC); summary of its role and reporting structure; statement of whether it is independent from the sponsor and competing interests; and reference to where further details about its charter can be found, if not in the protocol. Alternatively, an explanation of why a DMC is not needed	**Page 9**
	21b	Description of any interim analyses and stopping guidelines, including who will have access to these interim results and make the final decision to terminate the trial	**Page 9**
Harms	22	Plans for collecting, assessing, reporting, and managing solicited and spontaneously reported adverse events and other unintended effects of trial interventions or trial conduct	**Page 9**
Auditing	23	Frequency and procedures for auditing trial conduct, if any, and whether the process will be independent from investigators and the sponsor	**Page 9**

For peer review only - http://bmjopen.bmj.com/site/about/guidelines.xhtml

Ethics and dissemination

Research ethics approval	24	Plans for seeking research ethics committee/institutional review board (REC/IRB) approval	**Page 12**
Protocol amendments	25	Plans for communicating important protocol modifications (eg, changes to eligibility criteria, outcomes, analyses) to relevant parties (eg, investigators, REC/IRBs, trial participants, trial registries, journals, regulators)	**Page 12**
Consent or assent	26a	Who will obtain informed consent or assent from potential trial participants or authorised surrogates, and how (see Item 32)	**Page 12**
	26b	Additional consent provisions for collection and use of participant data and biological specimens in ancillary studies, if applicable	**n/a**
Confidentiality	27	How personal information about potential and enrolled participants will be collected, shared, and maintained in order to protect confidentiality before, during, and after the trial	**Page 12**
Declaration of interests	28	Financial and other competing interests for principal investigators for the overall trial and each study site	**Page 12**
Access to data	29	Statement of who will have access to the final trial dataset, and disclosure of contractual agreements that limit such access for investigators	**Page12**
Ancillary and post-trial care	30	Provisions, if any, for ancillary and post-trial care, and for compensation to those who suffer harm from trial participation	**Not included**
Dissemination policy	31a	Plans for investigators and sponsor to communicate trial results to participants, healthcare professionals, the public, and other relevant groups (eg, via publication, reporting in results databases, or other data sharing arrangements), including any publication restrictions	**Page 12**
	31b	Authorship eligibility guidelines and any intended use of professional writers	**Page 12**
	31c	Plans, if any, for granting public access to the full protocol, participant-level dataset, and statistical code	Not included

Appendices

For peer review only - http://bmjopen.bmj.com/site/about/guidelines.xhtml

Informed consent materials	32	Model consent form and other related documentation given to participants and authorised surrogates	**Documents attached to sumission**
Biological specimens	33	Plans for collection, laboratory evaluation, and storage of biological specimens for genetic or molecular analysis in the current trial and for future use in ancillary studies, if applicable	**n/a**

*It is strongly recommended that this checklist be read in conjunction with the SPIRIT 2013 Explanation & Elaboration for important clarification on the items. Amendments to the protocol should be tracked and dated. The SPIRIT checklist is copyrighted by the SPIRIT Group under the Creative Commons "Attribution-NonCommercial-NoDerivs 3.0 Unported" license.

For peer review only

BMJ Open

The CELEB trial: Comparative Effectiveness of Lung volume reduction surgery for Emphysema and Bronchoscopic lung volume reduction with valve placement. A protocol for a randomised controlled trial

Journal:	*BMJ Open*
Manuscript ID	bmjopen-2017-021368.R2
Article Type:	Protocol
Date Submitted by the Author:	31-Jul-2018
Complete List of Authors:	Buttery, Sara; Royal Brompton and Harefield NHS Foundation Trust,<br>Kemp, Samuel; Royal Brompton and Harefield NHS Foundation Trust<br>Shah, Pallav; Royal Brompton and Harefield NHS Foundation Trust<br>Waller, David; Barts Health NHS Trust<br>Jordan, Simon; Royal Brompton and Harefield NHS Foundation Trust<br>Lee, John; National University of Singapore,<br>Banya, Winston; Royal Brompton and Harefield NHS Foundation Trust<br>Steiner, Michael; Institute for Lung Health,<br>Hopkinson, Nicholas; Royal Brompton Hospital, Respiratory Muscle Lab
**Primary Subject Heading**:	Respiratory medicine
Secondary Subject Heading:	Health economics, Surgery
Keywords:	COPD, Lung Volume Reduction, Thoracic surgery < SURGERY, Emphysema < THORACIC MEDICINE

SCHOLARONE™
Manuscripts

The CELEB trial: Comparative Effectiveness of Lung volume reduction surgery for Emphysema and Bronchoscopic lung volume reduction with valve placement.

A protocol for a randomised controlled trial

[1]Sara Buttery, [1]Samuel V. Kemp, [1]Pallav L. Shah [3] David Waller, [1]Simon Jordan, [4]J T. Lee, [1]W. Banya, [2]Michael Steiner, *[1]Nicholas S. Hopkinson

[1]NIHR Respiratory Disease, Biomedical Research Unit at the Royal Brompton and Harefield NHS Foundation Trust and Imperial College London, London, UK

[2] Leicester Respiratory Biomedical Research Unit, University Hospitals of Leicester NHS Foundation Trust, Leicester, UK

[3] Barts Health NHS Trust London, London, UK

[4] National University of Singapore, Singapore, SG119260

Corresponding author: Nicholas S Hopkinson

Royal Brompton Hospital Campus, Imperial College London

Fulham Road

London

SW3 6HP

Email: n.hopkinson@ic.ac.uk

Telephone: 0207 351 8029

Fax: 0207 351 8939

For peer review only

Abstract:

Introduction: Although lung volume reduction surgery and bronchoscopic lung volume reduction with endobronchial valves have both been shown to improve lung function, exercise capacity and quality of life in appropriately selected patients with emphysema, there are no direct comparison data between the two procedures to inform clinical decision making.

Methods and Analysis: We describe the protocol of the CELEB study (**ISRCTN**19684749), a randomised controlled trial which will compare outcomes at 1 year between the two procedures, using a composite disease severity measure, the iBODE score, which includes body mass index, lung function, breathlessness and exercise capacity.

Ethics and Dissemination: Ethical approval to conduct the study has been obtained from the Fulham Research Ethics Committee, London (16/LO/0286). The outcome of this trial will provide information to guide treatment choices in this population and will be presented at national and international meetings and published in peer-reviewed journals. We will also disseminate the main results to all participants in a letter.

For peer review only

Strengths and Limitations

- This is a randomised controlled trial
- There is a strict patient selection criterion for this trial based on previous research.
- Currently there is equipoise between the two interventions being compared.
- Due to the nature of the interventions it is not possible to double blind the trial.
- It may be difficult to obtain longer term outcomes as patients may cross over after the 12 month period of the trial.

For peer review only - http://bmjopen.bmj.com/site/about/guidelines.xhtml

Introduction:

Chronic obstructive pulmonary disease (COPD) is a common and disabling condition which is now the third largest cause of death worldwide[1]. Breathlessness, exercise limitation and mortality in COPD are all associated with increased lung volumes occurring due to airflow obstruction and increased lung complicance[2]. The condition is progressive and despite optimum care including smoking cessation, pharmacotherapy and pulmonary rehabilitation, many patients remain breathless and limited in everyday activities[3 4]. Surgical and bronchoscopic approaches to lung volume reduction are available which can bring substantial benefits in appropriately selected individuals, though both are also associated with some risk.

Lung volume reduction surgery (LVRS) involves removing the worst affected area of emphasematous lung, which means that the remaining healthier and less compliant lung can function more effectively, with the respiratory muscles working at less of a mechanical discadvantages[5]. LVRS has been shown to improve survival, exercise capacity and quality of life in appropriately selected patients with heterogeneous emphysema and poor exercise capacity[5-9]. and is therefore recommended in national and international guidelines for the management of COPD[1 10 11]. However, uptake has been limited, due in part to exaggerated concerns about surgical morbidity and mortality[12]. In modern clinical practice morbidity and mortality from the procedure is substantially lower[9 13] than was the case in trials conducted around the turn of the century[5 6].

An alternative approach to resecting the target area of lung is to place endobronchial valves occluding the airways supplying a particular lobe. This form of bronchoscopic lung volume reduction (BLVR) can produce lobar atelectasis, and is intended to achieve similar benefits to LVRS but with less morbidity[14-24]. The approach is only effective in the absence of

For peer review only - http://bmjopen.bmj.com/site/about/guidelines.xhtml

interlobar collateral ventilation (CV)[23 25]. If this is present air can enter the target lobe from an adjacent lobe and atelectasis does not occur. In patients with a heterogenous pattern of emphysema and intact interlobar fissures, valve placement can produce significant improvements in lung function, exercise capacity and health status[23 26-28]. Yet complications do occur, in particular pneumothorax which occurs in up to 30% of cases[28] and can on occasion be fatal, as well as exacerbation-like events. Valve expectoration or misplacement can necessitate additional procedures.

Patients with heterogeneous emphysema and intact interlobar fissures, may benefit from either BLVR or LVRS, but there are no direct comparison data on the relative value of the two procedures to guide clinical decision making.

The CELEB study is a multi-centre, randomised – controlled parallel group trial that opened for recruitment in October 2016. Patients, who are considered to be suitable candidates for both forms of targeted lung reduction therapy, will be randomised to either BLVR or LVRS. The primary aim of CELEB study is to determine wether LVRS procedures a greater health benefit at one year than BLVR. This will be determined using change in the iBODE index, a composite meansure of COPD severity (made up of BMI, lung function, breathlessness and exercise capacity)[29]. Change in BODE score has been found to be a good predictor of survival in LVRS patients[30]. Health resource ultisation will also be compared between treatment arms. Clinical efficacy will be assessed over a 12 month period. The study is set up as a superiority trial and will be conducted, analysed and reported according to the Consolidation Standards of Reporting Trials (CONSORT) statement for randomised controlled trials. This paper will outline the protocol for the CELEB study (version 2.0, 19th February 2016).

For peer review only - http://bmjopen.bmj.com/site/about/guidelines.xhtml

Methods and Analysis:

Participants: Patients will be recruited from outpatient clinics at UK hospital sites which have an established multi-disciplinary team (MDT) meeting dedicated to identifying suitable candidates for LVR[31]. Patients will be eligible for the study if they fit the following criteria; significant airflow obstruction (FEV_1<60% predicted), limiting breathlessness (MRC dyspnoea score > 3), significant hyperinflation (TLC>100% predicted, RV>170% predicted) and are considered, using CT and perfusion scan, to have a heterogeneous emphysema pattern with intact interlobar fissures (>90%). They must also not have smoked for at least 3 months. Patients fitting these criteria will be identified from outpatient clinics and put forward for discussion at the MDT meeting. Patients will be excluded if they have pulmonary fibrosis or any other major co-morbidity that could affect survival or mean that LVR procedures are unlikely to be effective. Patients with PaO_2<7.0kPa and/or $PaCO_2$>7.0kPa will also be excluded. The MDT will make a decision on whether a patient is suitable for both interventions and if so, that there is equipoise between the treatments options. It is only after this is agreed that the screening visit will be arranged and consent taken.

Interventions: LVRS will be carried out by a thoracic surgeon under general anaesthetic using a unilateral VATS approach. The precise operation will be at the discretion of the surgeon, intended to remove the most emphysematous area of lung, leaving the best quality lung behind and minimising air leaks and other complications. Patients will initially go to the High Dependency Unit (HDU) post operatively and post-operative management will include attention to wound discomfort, management of chest drains and prompt mobilisation. Patient management, discharge and initial follow up will be determined by their clinical team.

For peer review only - http://bmjopen.bmj.com/site/about/guidelines.xhtml

BLVR will be performed via bronchoscopy by an operator experienced in placing EBV. The procedure will be performed under conscious sedation or general anaesthetic if necessary, with endobronchial valves placed to occlude the target lobe. A chest x-ray will be performed one hour post procedure and participants will spend three nights post-procedure as an in-patient routinely in case a pneumothorax occurs. They will be discharged with written advice about the signs of pneumothorax and what to do if these occur. Procedures including valve adjustment and replacement will be permitted to ensure treatment is optimised.

Participants will be followed up in clinic routinely about three months after discharge. Other clinic attendances will depend on clinical need with further investigations and procedures determined by their responsible clinician. Patients will receive a phone call from the study team at one month and at 6 and 9 months. There will be a final clinic visit at 12 months. A summary of the trial assessment is shown in **Table 1.**

Outcomes: The Primary outcome will be the change in iBODE score[32] at 12 months from baseline. The iBODE incorporates the incremental shuttle walk test, BMI, FEV_1 and the MRC dyspnoea score. Possible scores range from 0 to 10 with 10 with increasing scores associated with worsening mortality. The primary endpoint measures will be performed by staff blind to treatment allocation and patients will be asked not to reveal this in order to reduce bias.

Secondary outcomes will be assessed at baseline, 3 months and 12 months. Changes in respiratory related health status (COPD assessment test (CAT)), health related quality of life (HRQOL) using the EQ-5D-5L, residual volume (RV) and fat free mass (FFM) will be evaluated. Physical activity will be measured using the Proactive c-PPAC score.

The basis for the sample size calculation is the paper by Imfeld *et al* which addressed the response to LVRS[30]. This compared the improvement in BODE score at three months post LVRS between survivors and non-survivors at 5 years. Long term survivors had an improvement of 3.9 points at three months, the non–survivors, had an improvement of 2.4

For peer review only - http://bmjopen.bmj.com/site/about/guidelines.xhtml

points (*i.e.* a difference of 1.5 points). This difference is likely therefore to represent a clinically important difference between outcomes in the two LVR arms, being of sufficient magnitude to influence treatment decisions and is therefore a meaningful basis on which to power the study.

The study is powered on the basis of an average response to LVRS that will be the same as in the Imfeld paper (*i.e.* a fall of 3.1) and for BLVR that the improvement will be 1.6 (1.5 points less benefit). For practical purposes we have taken the BODE and the iBODE to be equivalent[29] and so, assuming an SD of 1.8 for the change in BODE score and taking a 5% significance level and 90% statistical power, 34 patients would be required in each treatment arm. Allowing for 10% drop-outs we will enrol 76 individuals.

Assignment of interventions: All participants will undergo a fibreoptic bronchoscopy to allow for assessment of the presence of collateral ventilation using the Chartis™ system[25] This uses a balloon catheter, inflated in the target airway, with a flow sensor and a distal pressure meter. Pressure swings should continue with inspiration and expiration, but flow will diminish and then cease as the target lobe empties if there is no collateral ventilation (CV –ve). If collateral ventilation is present, flow will continue (CV +ve). Subsequent participation in the trial depends on this. As the absence of collateral ventilation in the target lobe is inclusion criteria, patients with collateral ventilation are deemed to be screen failures and cannot proceed to randomisation (as they would not benefit from valve placement so there is no longer equipoise). However they will still be suitable for a surgical approach and may go on to "open label" LVRS. We will endeavour to follow these patients up at 12 months and their iBODE score will be evaluated. In some cases CV cannot be assessed either because there is continuing low flow or an abrupt cessation in the flow trace suggesting obstruction. It may be possible to work round this by measuring the presence or absence of CV in the adjacent lobe (*i.e.* the reciprocal position). Where CV is truly indeterminate, patients will be withdrawn from the study (*i.e.* treated as CV positive).

For peer review only - http://bmjopen.bmj.com/site/about/guidelines.xhtml

If the person has a CV negative lobar target for treatment they will be immediately allocated randomly to one of the treatment arms, using an online system. Randomisation will be on a 1:1 basis based on a computer generated random sequence with random block sizes generated by Sealed Envelope ltd (London, UK). This will include stratification by site and by iBODE score to ensure matched numbers in both arms with an iBODE score of ≥ 7.

Patients, the study co-ordinator and those providing clinical care will not be blind to treatment allocation. The assessors recording the components of the iBODE score will be blinded to reduce the potential for bias. Therefore, no un-blinding process will be necessary.

Data collection, management and analysis:

At baseline a full medical history including exacerbations, hospital admissions and drug history will be taken. Pulmonary Function Tests (spirometry, plethysmography and gas transfer measurements) will be carried out according to the American Thoracic Society/European Respiratory Society recommendations, together with arterial blood gas analysis. Additional measures will include Medical Research Council dyspnoea score, health status (CAT), HRQOL (EQ-5D-5L) and fat free mass using bioimpedance. Two incremental shuttle walk tests (ISWT) will be carried out due to the learning effect seen following a test walk[32] Figure 1 provides a summary of the trial design.

Phyiscal activity will be assessed using the PROactive Consortium patient reported outcome tool for physical activity in COPD[33-35]. Participants wear a Dynaport Movemonitor accelerometer for one week as a measure of physical activity at the end of this they complete the c-PACC physical activity questionnaire which asks about the amount and difficulty of physical activity during the period covered by the monitor.

A cost-utility analysis will be carried out from a National Health Service (NHS) perspective using data from the trial. The relative value of the two approaches will be based on

For peer review only - http://bmjopen.bmj.com/site/about/guidelines.xhtml

procedural cost, length of stay, days in hospital subsequently expressed as a cost per quality-adjusted life year (QALY) together with data gathered using the Client Socio-Demographic and Service Receipt Inventory (CSRI) which asks participants about their use of health and social services over the last three months. This will be completed at 3, 6, 9 and 12 months. They will be asked to report the frequency and intensity of their service use. Cost incurred in both arms may include (i) medical and surgical services received as an in-patient (ii) health care visits in relation to COPD (iii) COPD related medication use. Total cost of individuals will be estimated by multiplying resources use with national tariff used in the NHS. QALY will be estimated using EQ-5D-5L questionnaire. The five attributes of this questionnaire (mobility, self-care, usual activities, pain/discomfort/ anxiety/depression) will be summarised into a single UK-derived preference-based utility score.

Figure 1

Outcome data analysis will be by intention to treat. Patients with missing data will be assumed to have had zero change from baseline. Unpaired t-tests will be used to compare mean response across the 2 groups: control and intervention. Mixed linear model procedure to evaluate the effects of predefined covariables on dependent outcome variables.

Total costs will be calculated as the sum-product of resource use and unit cost for each patient. Incremental cost-effectiveness ratios (ICER) will be obtained by calculating the incremental costs divided by the incremental effects for the LVRS vs the BLVR groups`. Multivariate regression analysis will be used to adjust for baseline differences in health status between the trial groups. Uncertainty in the incremental cost-effectiveness ratio

For peer review only - http://bmjopen.bmj.com/site/about/guidelines.xhtml

(ICER) will be addressed using bootstrapping and the estimation of cost-effectiveness acceptability curves. Estimates of ICERs will be compared with the £20,000 to £30,000 per QALY threshold of cost-effectiveness recommended by the National Institute for Health and Care Excellence (NICE). No interim analyses are planned and the trial has no formal stopping guidelines.

Patient and public involvement

Prior to commencement of the trial we held two focus groups of patients with COPD who have undergone LVRS and/or BLVR. This allowed us to capture qualitative information about issues of importance to this group of patients beyond those captured by generic or disease specific health status tools. Participants will also be invited to help with reviewing dissemination materials.

For peer review only

For peer review only - http://bmjopen.bmj.com/site/about/guidelines.xhtml

.

Ethics and dissemination: Ethical approval to conduct the study has been obtained from the Fulham Research Ethics Committee, London (16/LO/0286)

Trial procedures will only begin following a clinical decision by the MDT that a patient is eligible for BLVR or LVRS. Patients will then receive verbal and written information regarding the study and informed written consent will be taken at the screening visit prior to any measurements being taken. A trial steering committee will provide overall supervision of the trial and ensures that it is being conducted in accordance with the principles of Good Clinical Practice (GCP) standards.

Contributorship Statements: This protocol was written by the Chief Investigator (CI) Nicolas Hopkinson (NSH) in accordance with the Sponsor's guidance for writing non-CTIMP protocols. NSH and the Research Office discussed and agreed this study protocol. All other applicants SB, SVK, PLS, DW, SJ, JLL, WB, MS were involved in trial design and revisions of the final protocol and will be involved in the production of the final manuscript upon trial completion. All investigators (NSH, SB, SVK, PLS, DW, SJ, MS) agreed to perform the investigations outlined in the study protocol and to abide by this protocol except in the case of medical emergency that will be notified to the Research Office. All authors have agreed upon the final manuscript for this submission.

We would also like to thank the patient advisors that attended the focus groups and contributed to the design of this study.

For peer review only - http://bmjopen.bmj.com/site/about/guidelines.xhtml

Funding Statement: The study is funded by a grant to Royal Brompton and Harefield NHS Foundation Trust, from the NIHR, through the Research for Patient Benefit scheme (PB-PG-1014-35051). Imperial College, London will support the reporting of this manuscript.

Competing Interests Statement: No support from any external organisation or financial relationships with any organisations that might have an interest in the submitted work has been sought for this study. Royal Brompton has received reimbursement of clinical trial expenses from PneumRx, PulmonX, Olympus, Uptake Medical, Holaira, and Creo Medical. The sponsor will not be involved in the conduct of the trial or influence any decision to publish.

Trial sponsor representative

Mr Patrik Pettersson

Royal Brompton and Harefield NHS Foundation Trust (RB&HFT)

Royal Brompton Hospital

Research office

Chelsea Wing, Level 2

Sydney Street

London , SW3 6NP

For peer review only

References

1. Lozano R, Naghavi M, Foreman K, et al. Global and regional mortality from 235 causes of death for 20 age groups in 1990 and 2010: a systematic analysis for the Global Burden of Disease Study 2010. *Lancet (London, England)* 2012;380(9859):2095-128. doi: 10.1016/s0140-6736(12)61728-0 [published Online First: 2012/12/19]

2. Celli BR, MacNee W. Standards for the diagnosis and treatment of patients with COPD: a summary of the ATS/ERS position paper. *The European respiratory journal* 2004;23(6):932-46. [published Online First: 2004/06/29]

3. Maddocks M, Lovell N, Booth S, et al. Palliative care and management of troublesome symptoms for people with chronic obstructive pulmonary disease. *Lancet (London, England)* 2017;390(10098):988-1002. doi: 10.1016/s0140-6736(17)32127-x [published Online First: 2017/09/06]

4. Kelly JL, Bamsey O, Smith C, et al. Health status assessment in routine clinical practice: the chronic obstructive pulmonary disease assessment test score in outpatients. *Respiration; international review of thoracic diseases* 2012;84(3):193-9. doi: 10.1159/000336549 [published Online First: 2012/03/24]

5. Criner GJ, Cordova F, Sternberg AL, et al. The National Emphysema Treatment Trial (NETT) Part II: Lessons learned about lung volume reduction surgery. *American journal of respiratory and critical care medicine* 2011;184(8):881-93. doi: 10.1164/rccm.201103-0455CI [published Online First: 2011/07/02]

6. Geddes D, Davies M, Koyama H, et al. Effect of lung-volume-reduction surgery in patients with severe emphysema. *The New England journal of medicine* 2000;343(4):239-45. doi: 10.1056/nejm200007273430402 [published Online First: 2000/07/27]

7. Fishman A, Martinez F, Naunheim K, et al. A randomized trial comparing lung-volume-reduction surgery with medical therapy for severe emphysema. *The New England journal of medicine* 2003;348(21):2059-73. doi: 10.1056/NEJMoa030287 [published Online First: 2003/05/22]

8. Ferguson GT, Fernandez E, Zamora MR, et al. Improved exercise performance following lung volume reduction surgery for emphysema. *American journal of respiratory and critical care medicine* 1998;157(4 Pt 1):1195-203. doi: 10.1164/ajrccm.157.4.9705008 [published Online First: 1998/05/01]

For peer review only

For peer review only - http://bmjopen.bmj.com/site/about/guidelines.xhtml

9. Clark SJ, Zoumot Z, Bamsey O, et al. Surgical approaches for lung volume reduction in emphysema. *Clinical medicine (London, England)* 2014;14(2):122-7. doi: 10.7861/clinmedicine.14-2-122 [published Online First: 2014/04/10]

10. Chronic obstructive pulmonary disease in adults | Guidance and guidelines | NICE: NICE; 2018 [Available from: https://www.nice.org.uk/guidance/qs10.

11. GOLD 2017 Global Strategy for the Diagnosis, Management and Prevention of COPD - Global Initiative for Chronic Obstructive Lung Disease - GOLD 2018 [Available from: http://goldcopd.org/gold-2017-global-strategy-diagnosis-management-prevention-copd/.

12. McNulty W, Jordan S, Hopkinson NS. Attitudes and access to lung volume reduction surgery for COPD: a survey by the British Thoracic Society. *BMJ open respiratory research* 2014;1(1):e000023. doi: 10.1136/bmjresp-2014-000023 [published Online First: 2014/12/06]

13. Greening NJ, Vaughan P, Oey I, et al. Individualised risk in patients undergoing lung volume reduction surgery: the Glenfield BFG score. *The European respiratory journal* 2017;49(6) doi: 10.1183/13993003.01766-2016 [published Online First: 2017/06/03]

14. Yim AP, Hwong TM, Lee TW, et al. Early results of endoscopic lung volume reduction for emphysema. *The Journal of thoracic and cardiovascular surgery* 2004;127(6):1564-73. doi: 10.1016/j.jtcvs.2003.10.005 [published Online First: 2004/06/03]

15. Wood DE, McKenna RJ, Jr., Yusen RD, et al. A multicenter trial of an intrabronchial valve for treatment of severe emphysema. *The Journal of thoracic and cardiovascular surgery* 2007;133(1):65-73. doi: 10.1016/j.jtcvs.2006.06.051 [published Online First: 2007/01/03]

16. Wan IY, Toma TP, Geddes DM, et al. Bronchoscopic lung volume reduction for end-stage emphysema: report on the first 98 patients. *Chest* 2006;129(3):518-26. doi: 10.1378/chest.129.3.518 [published Online First: 2006/03/16]

17. Venuta F, de Giacomo T, Rendina EA, et al. Bronchoscopic lung-volume reduction with one-way valves in patients with heterogenous emphysema. *The Annals of thoracic*

For peer review only

For peer review only - http://bmjopen.bmj.com/site/about/guidelines.xhtml

surgery 2005;79(2):411-6; discussion 16-7. doi: 10.1016/j.athoracsur.2004.07.048 [published Online First: 2005/02/01]

18. Toma TP, Hopkinson NS, Hillier J, et al. Bronchoscopic volume reduction with valve implants in patients with severe emphysema. *Lancet (London, England)* 2003;361(9361):931-3. doi: 10.1016/s0140-6736(03)12762-6 [published Online First: 2003/03/22]

19. Snell GI, Holsworth L, Borrill ZL, et al. The potential for bronchoscopic lung volume reduction using bronchial prostheses: a pilot study. *Chest* 2003;124(3):1073-80. [published Online First: 2003/09/13]

20. Hopkinson NS, Toma TP, Hansell DM, et al. Effect of bronchoscopic lung volume reduction on dynamic hyperinflation and exercise in emphysema. *American journal of respiratory and critical care medicine* 2005;171(5):453-60. doi: 10.1164/rccm.200407-961OC [published Online First: 2004/12/08]

21. Hopkinson NS, Kemp SV, Toma TP, et al. Atelectasis and survival after bronchoscopic lung volume reduction for COPD. *The European respiratory journal* 2011;37(6):1346-51. doi: 10.1183/09031936.00100110 [published Online First: 2010/10/16]

22. de Oliveira HG, Macedo-Neto AV, John AB, et al. Transbronchoscopic pulmonary emphysema treatment: 1-month to 24-month endoscopic follow-up. *Chest* 2006;130(1):190-9. doi: 10.1378/chest.130.1.190 [published Online First: 2006/07/15]

23. Davey C, Zoumot Z, Jordan S, et al. Bronchoscopic lung volume reduction with endobronchial valves for patients with heterogeneous emphysema and intact interlobar fissures (the BeLieVeR-HIFi study): a randomised controlled trial. *Lancet (London, England)* 2015;386(9998):1066-73. doi: 10.1016/s0140-6736(15)60001-0 [published Online First: 2015/06/28]

24. Davey C, Zoumot Z, Jordan S, et al. Bronchoscopic lung volume reduction with endobronchial valves for patients with heterogeneous emphysema and intact interlobar fissures (the BeLieVeR-HIFi trial): study design and rationale. *Thorax* 2015;70(3):288-90. doi: 10.1136/thoraxjnl-2014-205127 [published Online First: 2014/03/26]

For peer review only - http://bmjopen.bmj.com/site/about/guidelines.xhtml

25. Herth FJ, Eberhardt R, Gompelmann D, et al. Radiological and clinical outcomes of using Chartis to plan endobronchial valve treatment. *The European respiratory journal* 2013;41(2):302-8. doi: 10.1183/09031936.00015312 [published Online First: 2012/05/05]

26. Zoumot Z, Davey C, Jordan S, et al. Endobronchial valves for patients with heterogeneous emphysema and without interlobar collateral ventilation: open label treatment following the BeLieVeR-HIFi study. Thorax. England: Published by the BMJ Publishing Group Limited. For permission to use (where not already granted under a licence) please go to http://www.bmj.com/company/products-services/rights-and-licensing/. 2017:277-79.

27. Klooster K, ten Hacken NH, Hartman JE, et al. Endobronchial Valves for Emphysema without Interlobar Collateral Ventilation. *The New England journal of medicine* 2015;373(24):2325-35. doi: 10.1056/NEJMoa1507807 [published Online First: 2015/12/10]

28. Kemp SV, Slebos DJ, Kirk A, et al. A Multicenter Randomized Controlled Trial of Zephyr Endobronchial Valve Treatment in Heterogeneous Emphysema (TRANSFORM). *American journal of respiratory and critical care medicine* 2017;196(12):1535-43. doi: 10.1164/rccm.201707-1327OC [published Online First: 2017/09/09]

29. Williams JE, Green RH, Warrington V, et al. Development of the i-BODE: validation of the incremental shuttle walking test within the BODE index. *Respiratory medicine* 2012;106(3):390-6. doi: 10.1016/j.rmed.2011.09.005 [published Online First: 2011/10/08]

30. Imfeld S, Bloch KE, Weder W, et al. The BODE index after lung volume reduction surgery correlates with survival. *Chest* 2006;129(4):873-8. doi: 10.1378/chest.129.4.873 [published Online First: 2006/04/13]

31. Rathinam S, Oey I, Steiner M, et al. The role of the emphysema multidisciplinary team in a successful lung volume reduction surgery programmedagger. *European journal of cardio-thoracic surgery : official journal of the European Association for Cardio-thoracic Surgery* 2014;46(6):1021-6; discussion 26. doi: 10.1093/ejcts/ezu129 [published Online First: 2014/04/29]

For peer review only - http://bmjopen.bmj.com/site/about/guidelines.xhtml

32. Hakamy A, McKeever TM, Steiner MC, et al. The use of the practice walk test in pulmonary rehabilitation program: National COPD Audit Pulmonary Rehabilitation Workstream. *International journal of chronic obstructive pulmonary disease* 2017;12:2681-86. doi: 10.2147/copd.s141620 [published Online First: 2017/09/22]

33. Dobbels F, de Jong C, Drost E, *et al*. The PROactive innovative conseptual framework on physical activity. *European Respiratory Journal* 2014;44(5):1223-33. doi: 10.1136/thoraxjnl-2013-204763

34. Gimeno-Santos E, Raste Y, Demeyer H, et al. The PROactive instruments to measure physical activity in patients with chronic obstructive pulmonary disease. *The European respiratory journal* 2015;46(4):988-1000. doi: 10.1183/09031936.00183014 [published Online First: 2015/05/30]

35. Gimeno-Santos E, Frei A, Steurer-Stey C, et al. Determinants and outcomes of physical activity in patients with COPD: a systematic review. *Thorax* 2014;69(8):731-9. doi: 10.1136/thoraxjnl-2013-204763 [published Online First: 2014/02/22]

Figure Legends

Figure 1: *schematic* outline of the trial design

For peer review only

For peer review only - http://bmjopen.bmj.com/site/about/guidelines.xhtml

Table 1: Summary of trial assessments

1 Spirometry, gas transfer, plethysmographic lung volumes, capillary blood gases

	Visit 1<br>Baseline	Visit 2<br>Chartis measurement	Visit 3<br>Admission for procedure	Visit 4<br>One month safety phone call	Visit 5<br>3 months follow up	6 months	9 months	Visit 6<br>1 year<br>Follow up
History, physical exam	x				x			
Informed consent	x							
FFM	x							x
MRC score	x				x			x
CAT score	x				x			x
Full pulmonary function[1]	x				x			x
CT thorax	x				x			
iSWT	x				x			x
Activity monitor given for 7 days	x							x<br>(7 days prior to visit)
Activity monitor collected		x						x
c-PPAC score		x						x
Bronchoscopy with Chartis measurement of collateral ventilation		x						
Randomisation		x						
Treatment intervention:<br>LVRS or BLVR			x					
Health resource utilisation<br>(CSRI questionnaire)					x	x	x	x

For peer review only

For peer review only - http://bmjopen.bmj.com/site/about/guidelines.xhtml

For peer review only

For peer review only - http://bmjopen.bmj.com/site/about/guidelines.xhtml

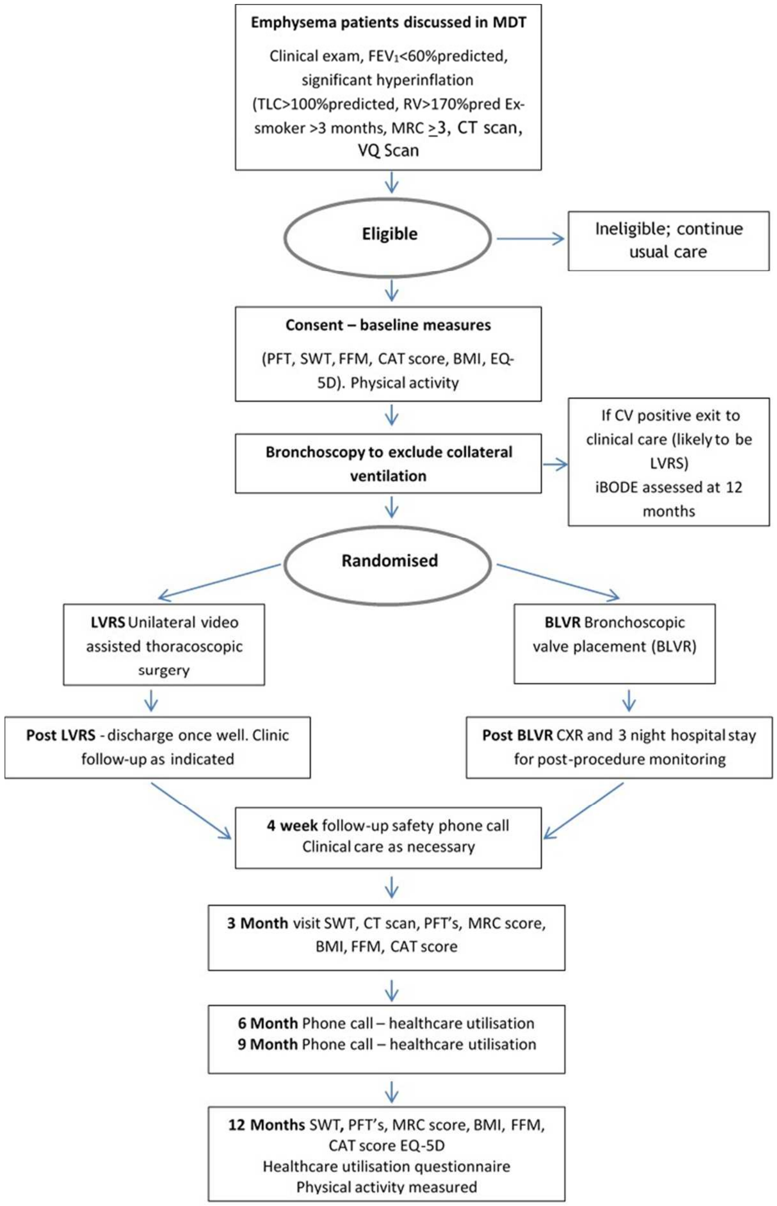

Figure 1

63x99mm (300 x 300 DPI)

SPIRIT

STANDARD PROTOCOL ITEMS: RECOMMENDATIONS FOR INTERVENTIONAL TRIALS

SPIRIT 2013 Checklist: Recommended items to address in a clinical trial protocol and related documents*

Section/item	Item No	Description	Addressed on page number
**Administrative information**			
Title	1	Descriptive title identifying the study design, population, interventions, and, if applicable, trial acronym	**page 1**
Trial registration	2a	Trial identifier and registry name. If not yet registered, name of intended registry	**Page 1: abstract**
	2b	All items from the World Health Organization Trial Registration Data Set	______________
Protocol version	3	Date and version identifier	**page 2: intro**
Funding	4	Sources and types of financial, material, and other support	**Page 13**
Roles and responsibilities	5a	Names, affiliations, and roles of protocol contributors	**Page 12: authors contributions**
	5b	Name and contact information for the trial sponsor	**Page 13**
	5c	Role of study sponsor and funders, if any, in study design; collection, management, analysis, and interpretation of data; writing of the report; and the decision to submit the report for publication, including whether they will have ultimate authority over any of these activities	**Page 13**

For peer review only

For peer review only - http://bmjopen.bmj.com/site/about/guidelines.xhtml

	5d	Composition, roles, and responsibilities of the coordinating centre, steering committee, endpoint adjudication committee, data management team, and other individuals or groups overseeing the trial, if applicable (see Item 21a for data monitoring committee)	**Page 13**
**Introduction**			
Background and rationale	6a	Description of research question and justification for undertaking the trial, including summary of relevant studies (published and unpublished) examining benefits and harms for each intervention	**Page 3/4**
	6b	Explanation for choice of comparators	**Page 3/4**
Objectives	7	Specific objectives or hypotheses	**Page 3/4**
Trial design	8	Description of trial design including type of trial (eg, parallel group, crossover, factorial, single group), allocation ratio, and framework (eg, superiority, equivalence, noninferiority, exploratory)	**Page 3/4**
**Methods: Participants, interventions, and outcomes**			
Study setting	9	Description of study settings (eg, community clinic, academic hospital) and list of countries where data will be collected. Reference to where list of study sites can be obtained	**Page 4/5**
Eligibility criteria	10	Inclusion and exclusion criteria for participants. If applicable, eligibility criteria for study centres and individuals who will perform the interventions (eg, surgeons, psychotherapists)	**Page 4/5**
Interventions	11a	Interventions for each group with sufficient detail to allow replication, including how and when they will be administered	**Page 5**
	11b	Criteria for discontinuing or modifying allocated interventions for a given trial participant (eg, drug dose change in response to harms, participant request, or improving/worsening disease)	**Page 5**
	11c	Strategies to improve adherence to intervention protocols, and any procedures for monitoring adherence (eg, drug tablet return, laboratory tests)	**Page 5**
	11d	Relevant concomitant care and interventions that are permitted or prohibited during the trial	**Page 5**

For peer review only - http://bmjopen.bmj.com/site/about/guidelines.xhtml

Outcomes	12	Primary, secondary, and other outcomes, including the specific measurement variable (eg, systolic blood pressure), analysis metric (eg, change from baseline, final value, time to event), method of aggregation (eg, median, proportion), and time point for each outcome. Explanation of the clinical relevance of chosen efficacy and harm outcomes is strongly recommended	**Page 6**
Participant timeline	13	Time schedule of enrolment, interventions (including any run-ins and washouts), assessments, and visits for participants. A schematic diagram is highly recommended (see Figure)	**Page 7**
Sample size	14	Estimated number of participants needed to achieve study objectives and how it was determined, including clinical and statistical assumptions supporting any sample size calculations	**Page 7**
Recruitment	15	Strategies for achieving adequate participant enrolment to reach target sample size	**Page 7**
**Methods: Assignment of interventions (for controlled trials)**			
Allocation:			
Sequence generation	16a	Method of generating the allocation sequence (e.g., computer-generated random numbers), and list of any factors for stratification. To reduce predictability of a random sequence, details of any planned restriction (eg, blocking) should be provided in a separate document that is unavailable to those who enrol participants or assign interventions	**Page 6**
Allocation concealment mechanism	16b	Mechanism of implementing the allocation sequence (eg, central telephone; sequentially numbered, opaque, sealed envelopes), describing any steps to conceal the sequence until interventions are assigned	**Page 6**
Implementation	16c	Who will generate the allocation sequence, who will enrol participants, and who will assign participants to interventions	**Page 6**
Blinding (masking)	17a	Who will be blinded after assignment to interventions (eg, trial participants, care providers, outcome assessors, data analysts), and how	**Page 6**
	17b	If blinded, circumstances under which unblinding is permissible, and procedure for revealing a participant's allocated intervention during the trial	**Page 6**

For peer review only - http://bmjopen.bmj.com/site/about/guidelines.xhtml

Methods: Data collection, management, and analysis

Data collection methods	18a	Plans for assessment and collection of outcome, baseline, and other trial data, including any related processes to promote data quality (eg, duplicate measurements, training of assessors) and a description of study instruments (eg, questionnaires, laboratory tests) along with their reliability and validity, if known. Reference to where data collection forms can be found, if not in the protocol	**Page 8**
	18b	Plans to promote participant retention and complete follow-up, including list of any outcome data to be collected for participants who discontinue or deviate from intervention protocols	**Page 8**
Data management	19	Plans for data entry, coding, security, and storage, including any related processes to promote data quality (eg, double data entry; range checks for data values). Reference to where details of data management procedures can be found, if not in the protocol	**Page 8/9**
Statistical methods	20a	Statistical methods for analysing primary and secondary outcomes. Reference to where other details of the statistical analysis plan can be found, if not in the protocol	**Page 8/9**
	20b	Methods for any additional analyses (eg, subgroup and adjusted analyses)	**Page 8/9**
	20c	Definition of analysis population relating to protocol non-adherence (eg, as randomised analysis), and any statistical methods to handle missing data (eg, multiple imputation)	**Page 8/9**

Methods: Monitoring

Data monitoring	21a	Composition of data monitoring committee (DMC); summary of its role and reporting structure; statement of whether it is independent from the sponsor and competing interests; and reference to where further details about its charter can be found, if not in the protocol. Alternatively, an explanation of why a DMC is not needed	**Page 9**
	21b	Description of any interim analyses and stopping guidelines, including who will have access to these interim results and make the final decision to terminate the trial	**Page 9**
Harms	22	Plans for collecting, assessing, reporting, and managing solicited and spontaneously reported adverse events and other unintended effects of trial interventions or trial conduct	**Page 9**
Auditing	23	Frequency and procedures for auditing trial conduct, if any, and whether the process will be independent from investigators and the sponsor	**Page 9**

For peer review only - http://bmjopen.bmj.com/site/about/guidelines.xhtml

Ethics and dissemination

Research ethics approval	24	Plans for seeking research ethics committee/institutional review board (REC/IRB) approval	**Page 12**
Protocol amendments	25	Plans for communicating important protocol modifications (eg, changes to eligibility criteria, outcomes, analyses) to relevant parties (eg, investigators, REC/IRBs, trial participants, trial registries, journals, regulators)	**Page 12**
Consent or assent	26a	Who will obtain informed consent or assent from potential trial participants or authorised surrogates, and how (see Item 32)	**Page 12**
	26b	Additional consent provisions for collection and use of participant data and biological specimens in ancillary studies, if applicable	**n/a**
Confidentiality	27	How personal information about potential and enrolled participants will be collected, shared, and maintained in order to protect confidentiality before, during, and after the trial	**Page 12**
Declaration of interests	28	Financial and other competing interests for principal investigators for the overall trial and each study site	**Page 12**
Access to data	29	Statement of who will have access to the final trial dataset, and disclosure of contractual agreements that limit such access for investigators	**Page12**
Ancillary and post-trial care	30	Provisions, if any, for ancillary and post-trial care, and for compensation to those who suffer harm from trial participation	**Not included**
Dissemination policy	31a	Plans for investigators and sponsor to communicate trial results to participants, healthcare professionals, the public, and other relevant groups (eg, via publication, reporting in results databases, or other data sharing arrangements), including any publication restrictions	**Page 12**
	31b	Authorship eligibility guidelines and any intended use of professional writers	**Page 12**
	31c	Plans, if any, for granting public access to the full protocol, participant-level dataset, and statistical code	Not included

Appendices

For peer review only - http://bmjopen.bmj.com/site/about/guidelines.xhtml

Informed consent materials	32	Model consent form and other related documentation given to participants and authorised surrogates	**Documents attached to sumission**
Biological specimens	33	Plans for collection, laboratory evaluation, and storage of biological specimens for genetic or molecular analysis in the current trial and for future use in ancillary studies, if applicable	**n/a**

*It is strongly recommended that this checklist be read in conjunction with the SPIRIT 2013 Explanation & Elaboration for important clarification on the items. Amendments to the protocol should be tracked and dated. The SPIRIT checklist is copyrighted by the SPIRIT Group under the Creative Commons "Attribution-NonCommercial-NoDerivs 3.0 Unported" license.

For peer review only
